# Supplementary material for: Validation and Comparison of Non-stationary Cognitive Models: A Diffusion Model Application
Source: Comput Brain Behav. 2024 Oct 8;8(2):191–210. doi: 10.1007/s42113-024-00218-4 (PMC13298658; doi:10.1007/s42113-024-00218-4)
Supplement: Supplementary file 1 — (pdf 11304 KB) [file 42113_2024_218_MOESM1_ESM.pdf]

Appendix A Prior Distributions

In the following we list the prior distributions we used for all four NSDDM's.

DDM Starting Values

For the starting values of the parameter trajectories we used half-normal distributions with a mean μ and a standard deviation σ denoted as $\mathcal{HN}(\mu, \sigma)$:

$$\begin{aligned} v_0 &\sim \mathcal{HN}(2.0, 2.0) \\ a_0 &\sim \mathcal{HN}(2.0, 1.5) \\ \tau_0 &\sim \mathcal{HN}(0.3, 1.0) \end{aligned}$$

Random Walk Transition Model

Half-normal distributions were used for the standard deviations of the Gaussian random walk transition model:

$$\begin{aligned} \sigma_v &\sim \mathcal{HN}(0.0, 0.1) \\ \sigma_a &\sim \mathcal{HN}(0.0, 0.1) \\ \sigma_\tau &\sim \mathcal{HN}(0.0, 0.01) \end{aligned}$$

We decided to use a relatively narrower prior on σ_τ because the non-decision time parameter is not expected to fluctuate as heavily as the other two parameters.

Mixture Random Walk Transition Model

The mixture random walk transition model used the same prior for the Gaussian random walk as described above. Additionally, Uniform distributions denoted as $\mathcal{U}$ were used for the mixture proportion parameter ρ:

$$\begin{aligned} \rho_v &\sim \mathcal{U}(0.0, 0.2) \\ \rho_a &\sim \mathcal{U}(0.0, 0.1) \end{aligned}$$

Lévy Flight Transition Model

The Lévy flight transition model uses an alpha stable distribution instead of a Gaussian distribution for the transition. We used the same priors for the standard deviations as in the random walk and the mixture random walk. The alpha stable distribution has an additional parameter α, which determines the fatness of the tails. This parameter is bound between 1 and 2. Therefore, we used a Beta distribution denoted as B and added 1 to the sampled values:

$$\begin{aligned} \tilde{\alpha}_v &\sim \mathrm{B}(1.5, 1.5) \\ \tilde{\alpha}_a &\sim \mathrm{B}(2.5, 1.5) \\ \alpha_v &= \tilde{\alpha}_v + 1 \\ \alpha_a &= \tilde{\alpha}_a + 1 \end{aligned}$$

Regime Switching Transition Model

The same prior distributions as for the mixture random walk were used for the mixture probabilities of the regime switching transition model.

Appendix B Neural Network Architectures and Training Setups

In the following, we outline our implementation of the neural approximators and the training setup used for model comparison and parameter estimation.

Model Comparison

For model comparison we trained an ensemble of ten neural approximators. Each approximator consists of a summary network and an inference network. The summary network is a many-to-one transformer architecture for time series encoding (Wen et al., 2023). The time series transformer has 128 template and 64 summary dimensions. For inference, we use a network that approximates posterior model probabilities (PMPs) as employed in Elsemüller, Schnuerch, et al. (2023).

We performed offline training for each of the ten neural approximators separately. The training data consisted of 25 000 simulations per model. Training was performed with 25 epochs and a batch size of 16 starting with an initial learning rate of 0.0005. The learning rate was adjusted with a cosine decay from its initial value to 0.

Parameter Estimation

For parameter estimation we trained one neural approximator for each of the four NSDDM implementations. Each approximator consists of a hierarchical summary network as employed in Elsemüller, Schnuerch, et al. (2023) and two inference networks. Three bidirectional long-short term memory (LSTM) networks were used for the hierarchical summary network. The number of hidden units were 512, 256, and 128 respectively.

For inference, we use a composition of two invertible neural networks (Radev, Mertens, et al., 2020), one for the low-level and one for the high-level parameters. The network for the low-level parameters has 8 coupling layers with an interleaved *affine* and *spline* internal coupling design. The network for the high-level parameters only differs from the former in its number of coupling layers which is 6.

Since our simulators can be run fast, the training of the four neural approximators was performed online, with 75 epochs, 1 000 iterations per epoch, and a batch size of 16. Thus, each approximator was trained on $N = 1\,200\,000$ simulated data sets. The initial learning rate was set to 0.0005 and was reduced with a cosine decay function to 0.

Appendix C Model Misspecification

The validation of our model comparison workflow indicated that the mixture random walk DDM and the regime switching DDM are often confused with each other. Therefore, we took a closer look at the comparison of their trajectories. First, we investigated their performance in the *closed world* by cross-fitting them on the basis of synthetic data. Second, we directly compared the parameter trajectories from both models inferred for each of the 14 participants separately (open world).

C.1 Closed World

We simulated 100 synthetic datasets, each consisting of $T = 800$ trials, using the mixture random walk DDM and the regime-switching model separately. We then fitted both models to both types of datasets (cross-fitting). To evaluate the models' parameter recovery performance, we calculated the normalized root mean squared error (NRMSE) between the true and estimated parameters across all time steps for both scenarios (i.e., well-specified and misspecified). The NRMSE is given by:

$$\mathrm{NRMSE}(\hat{\theta}, \theta) = \frac{\sqrt{\frac{1}{n}\sum_{i=1}^{n}(\theta_i - \hat{\theta}_i)^2}}{\hat{\theta}_{\max} - \hat{\theta}_{\min}},$$

where θ_i and $\hat{\theta}_i$ represent the true and estimated univariate parameter, respectively, and $\hat{\theta}_{\max}$ and $\hat{\theta}_{\min}$ are the maximum and minimum estimated parameter values used for normalization.

Fig C.1 shows the NRMSE for each model and parameter separately. We observed no notable difference between fitting a model to self-generated data (well-specified) and fitting it to data generated by the other model (misspecified). This suggests that both transition models can reproduce each other's trajectories quite well, making them difficult to distinguish in data space.

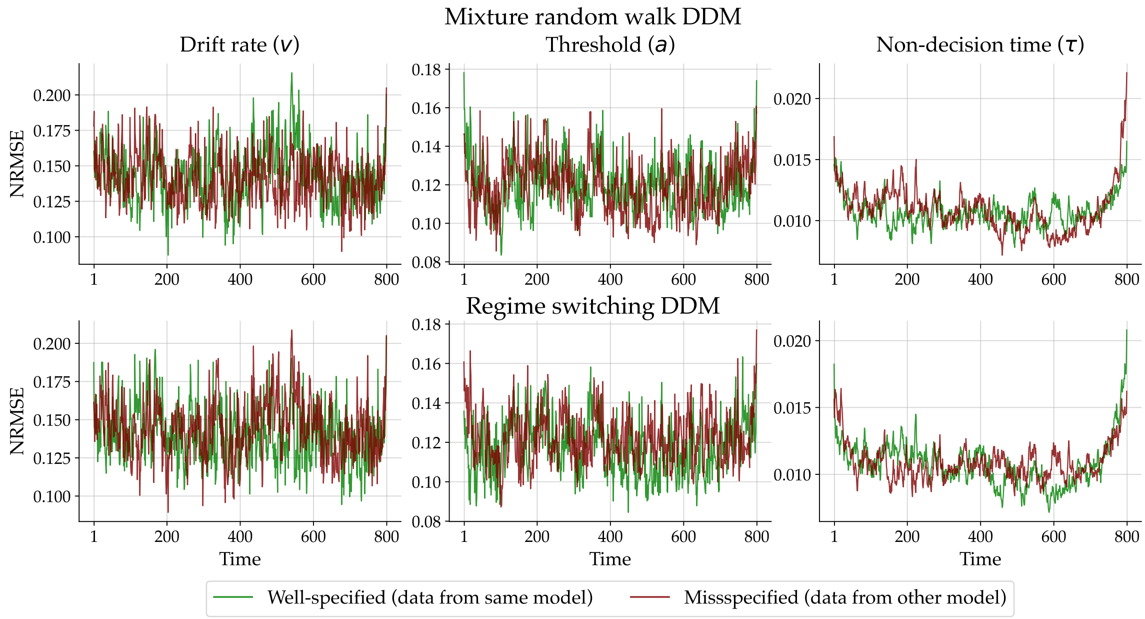

Fig. C1 The average parameter recovery performance for the mixture random walk DDM and the regime switching DDM measured by the normalized root mean squared error (NRSME) between true data-generating and estimated parameters. The green lines indicate the results in the well-specified scenarios where a model is fitted to data generated by the relative model. The red lines indicate the results in the missspecified scenarios where a model is fitted to the data simulated by the other model.

C.2 Open World

Fig C.2 shows the parameter trajectories inferred using the mixture random walk DDM (red lines) and the regime-switching model (green lines) for each subject and core DDM parameter. The estimates for the drift rate and non-decision time are nearly identical between the models. Similarly, the threshold parameter trajectories are consistent for most subjects. However, for a few subjects (e.g., subjects 2 and 9), the models diverge significantly. The cause of this discrepancy remains unclear, and is likely due to the superior expressiveness of the mixture random walk DDM on the real data.

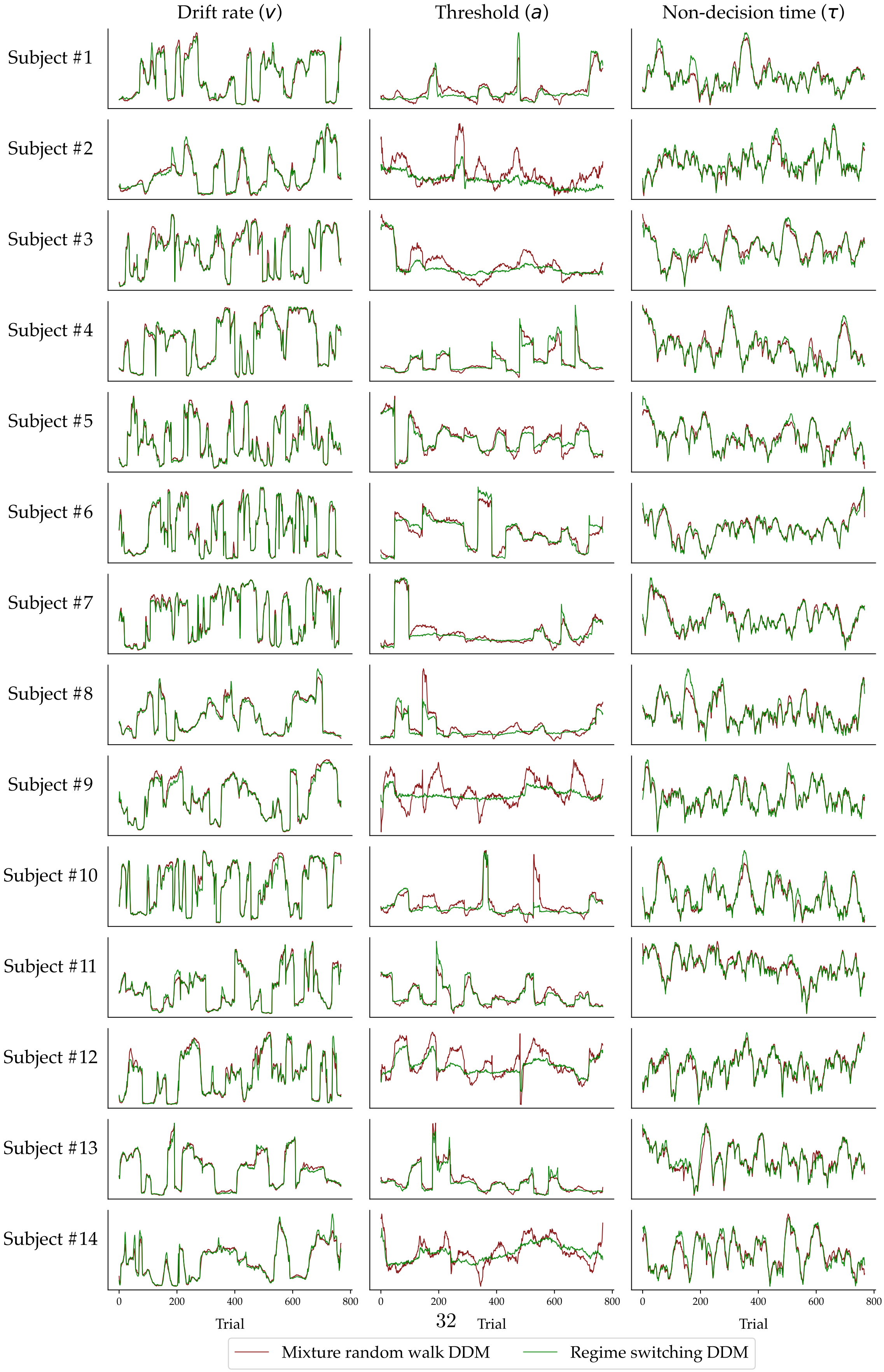

Fig. C2 The median parameter trajectories inferred with the mixture random walk DDM (red) and the regime switching DDM (green) for each of the 14 subject and core DDM parameter (drift rate, threshold, non-decision time) separately.

Appendix D Ablation Study

To assess the stability of our results, we conducted an ablation study. We fitted our models to subsets of the data ($1/8, 1/4$ and $1/2$ of the total trials per person). We then performed posterior re-simulations and evaluated the absolute fit to the data at an aggregated level, replicating Fig 6.

The absolute goodness-of-fit remains relatively strong even with as few as approximately $T = 100$ trials per person (Fig D). As expected, the uncertainty in posterior re-simulations and parameter estimates increases as the number of trials decreases. Nevertheless, the patterns remain robust with about 100 trials.

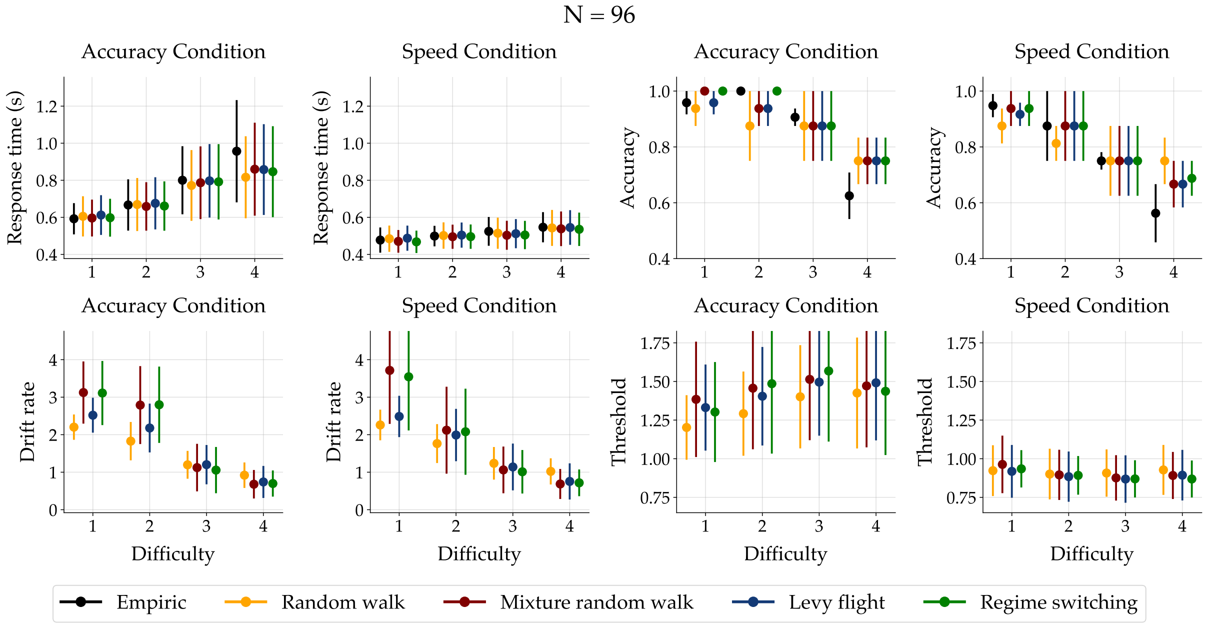

Fig. D3 Aggregated results from all models fitted to 1/8 of the total trials of the empirical data. The top row illustrates posterior re-simulations as a measure of the model's generative performance and absolute goodness-of-fit to the data. The bottom row depicts parameter estimates of the drift rate and the threshold parameter from the non-stationary diffusion decision models (NSDDM). **a** Empirical and re-simulated RTs for each difficulty level and both conditions. **b** Empirical and re-simulated proportions of correct choices (accuracy) for each difficulty level and both conditions separately. **c** Posterior estimates of the drift rate parameter for each difficulty level and both conditions separately. **d** Posterior estimates of the threshold parameter for each difficulty level and both conditions separately. Points indicate medians and the error bars represent the median absolute deviations (MAD) across individuals and re-simulations.

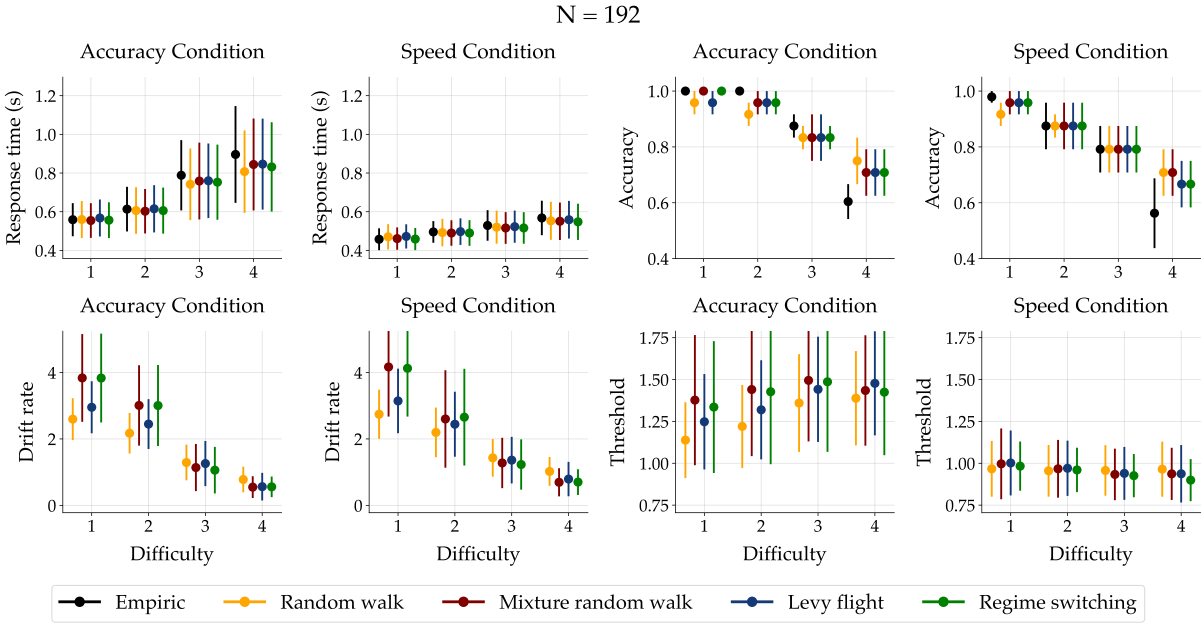

Fig. D4 Aggregated results from all models fitted to 1/4 of the total trials of the empirical data. The top row illustrates posterior re-simulations as a measure of the model's generative performance and absolute goodness-of-fit to the data. The bottom row depicts parameter estimates of the drift rate and the threshold parameter from the non-stationary diffusion decision models (NSDDM). **a** Empirical and re-simulated RTs for each difficulty level and both conditions. **b** Empirical and re-simulated proportions of correct choices (accuracy) for each difficulty level and both conditions separately. **c** Posterior estimates of the drift rate parameter for each difficulty level and both conditions separately. **d** Posterior estimates of the threshold parameter for each difficulty level and both conditions separately. Points indicate medians and the error bars represent the median absolute deviations (MAD) across individuals and re-simulations.

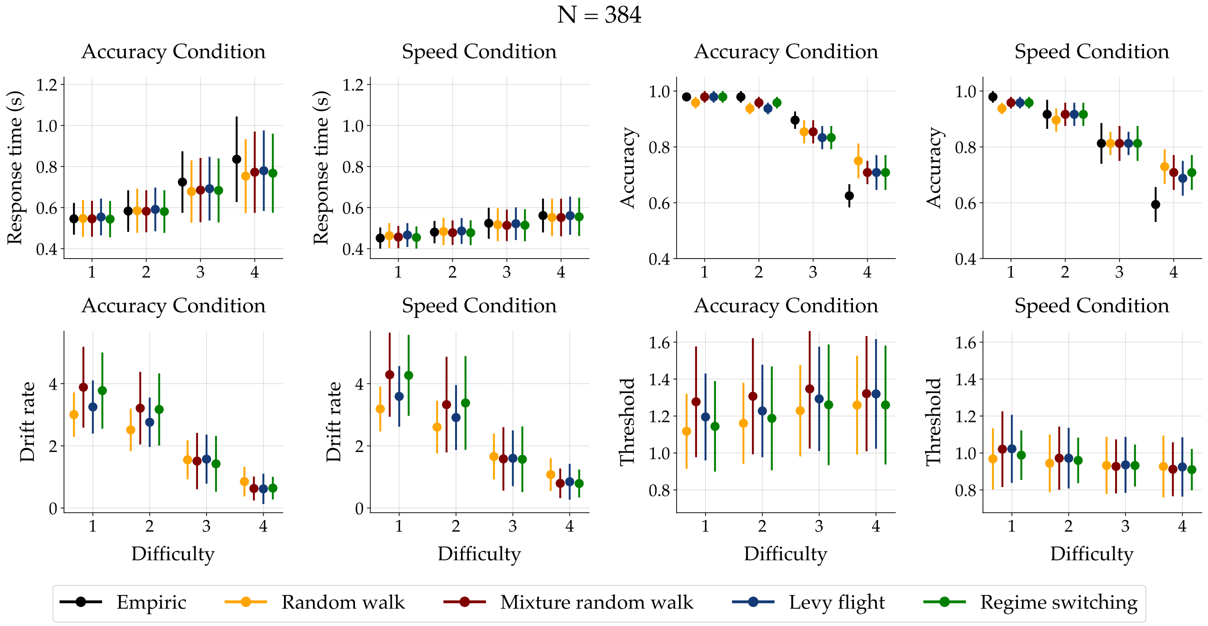

Fig. D5 Aggregated results from all models fitted to 1/2 of the total trials of the empirical data. The top row illustrates posterior re-simulations as a measure of the model's generative performance and absolute goodness-of-fit to the data. The bottom row depicts parameter estimates of the drift rate and the threshold parameter from the non-stationary diffusion decision models (NSDDM). **a** Empirical and re-simulated RTs for each difficulty level and both conditions. **b** Empirical and re-simulated proportions of correct choices (accuracy) for each difficulty level and both conditions separately. **c** Posterior estimates of the drift rate parameter for each difficulty level and both conditions separately. **d** Posterior estimates of the threshold parameter for each difficulty level and both conditions separately. Points indicate medians and the error bars represent the median absolute deviations (MAD) across individuals and re-simulations.

Appendix E Individual Analysis

The following section shows the individual specific posterior re-simulations and parameter estimates for each difficulty level and both conditions separately. The visualizations are constructed in the vain of Fig 6 in the main text.

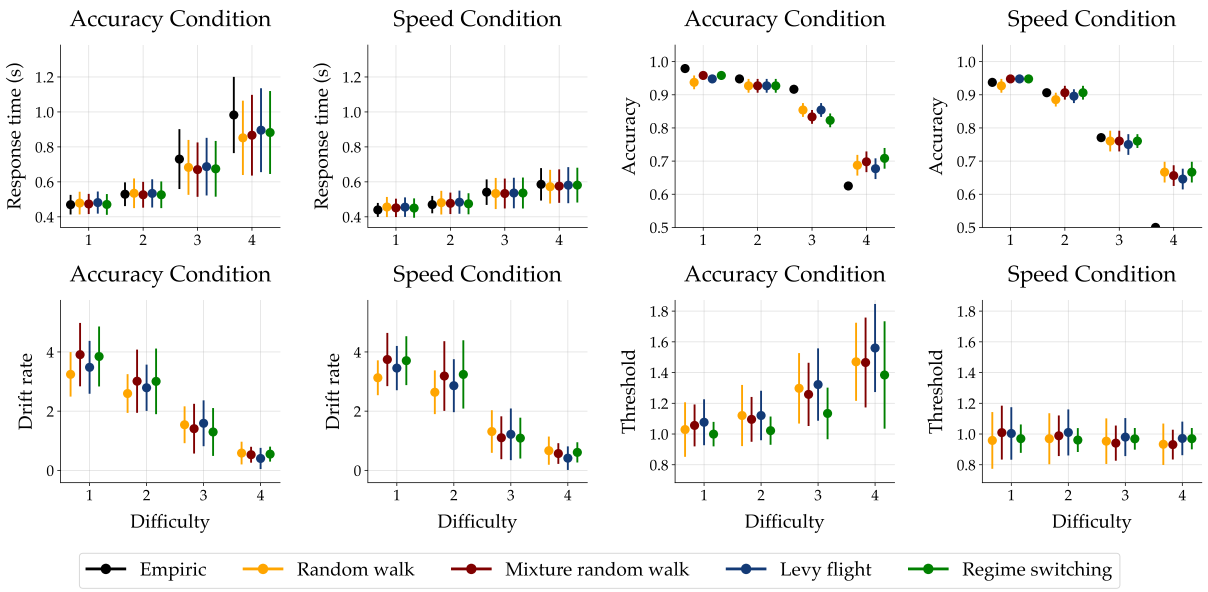

Fig. E6 Aggregate results from all models fitted to the data from participant 1. The top row illustrates posterior re-simulations as a measure of the model's generative performance and absolute goodness-of-fit to the data. The bottom row depicts parameter estimates of the drift rate and the threshold parameter from the non-stationary diffusion decision models (NSDDM). **a** Empirical and re-simulated response times for each difficulty level and both conditions. **b** Empirical and re-simulated proportions of correct choices (accuracy) for each difficulty level and both conditions separately. **c** Posterior estimates of the drift rate parameter for each difficulty level and both conditions separately. **d** Posterior estimates of the threshold parameter for each difficulty level and both conditions separately. Points indicate medians and the error bars represent the median absolute deviations (MAD) across individual data and re-simulations.

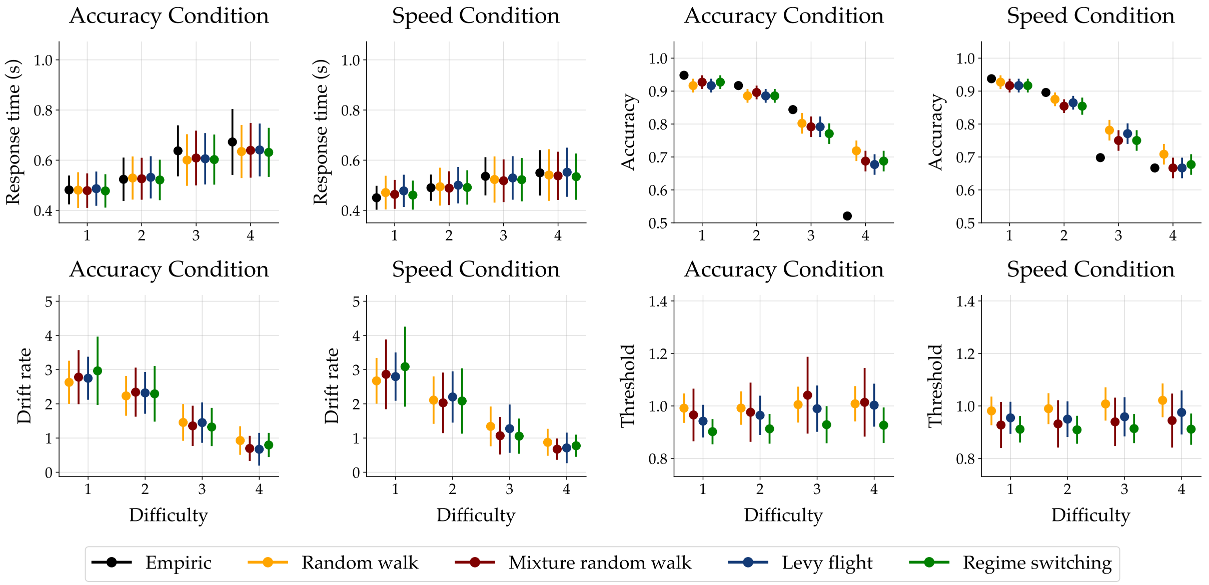

Fig. E7 Aggregate results from all models fitted to the data from participant 2. The top row illustrates posterior re-simulations as a measure of the model's generative performance and absolute goodness-of-fit to the data. The bottom row depicts parameter estimates of the drift rate and the threshold parameter from the non-stationary diffusion decision models (NSDDM). **a** Empirical and re-simulated response times for each difficulty level and both conditions. **b** Empirical and re-simulated proportions of correct choices (accuracy) for each difficulty level and both conditions separately. **c** Posterior estimates of the drift rate parameter for each difficulty level and both conditions separately. **d** Posterior estimates of the threshold parameter for each difficulty level and both conditions separately. Points indicate medians and the error bars represent the median absolute deviations (MAD) across individual data and re-simulations.

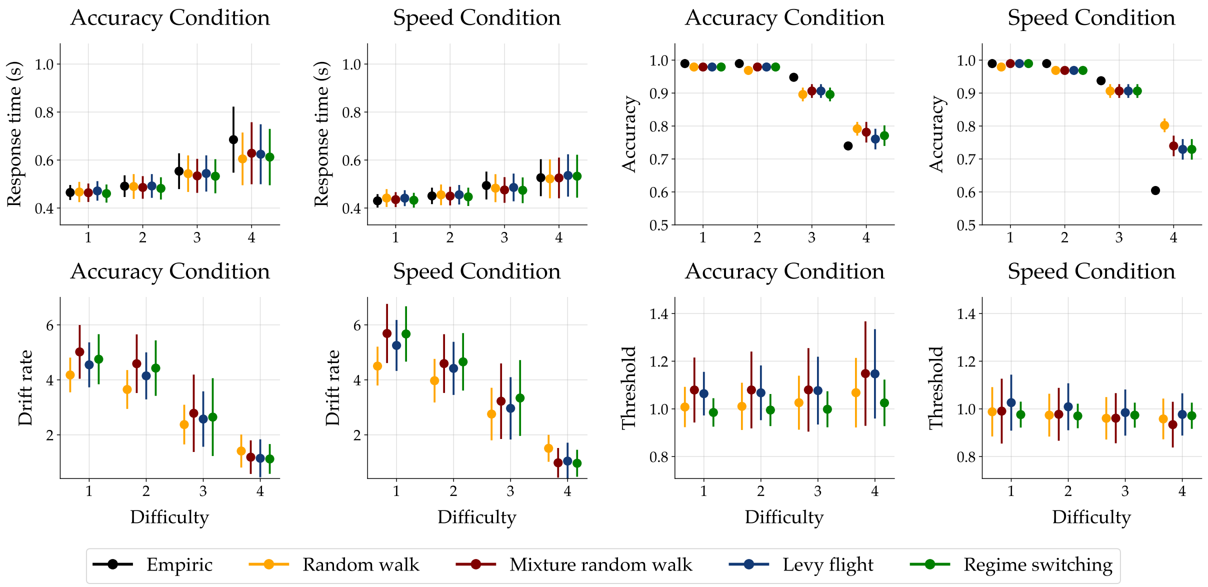

Fig. E8 Aggregate results from all models fitted to the data from participant 3. The top row illustrates posterior re-simulations as a measure of the model's generative performance and absolute goodness-of-fit to the data. The bottom row depicts parameter estimates of the drift rate and the threshold parameter from the non-stationary diffusion decision models (NSDDM). **a** Empirical and re-simulated response times for each difficulty level and both conditions. **b** Empirical and re-simulated proportions of correct choices (accuracy) for each difficulty level and both conditions separately. **c** Posterior estimates of the drift rate parameter for each difficulty level and both conditions separately. **d** Posterior estimates of the threshold parameter for each difficulty level and both conditions separately. Points indicate medians and the error bars represent the median absolute deviations (MAD) across individual data and re-simulations.

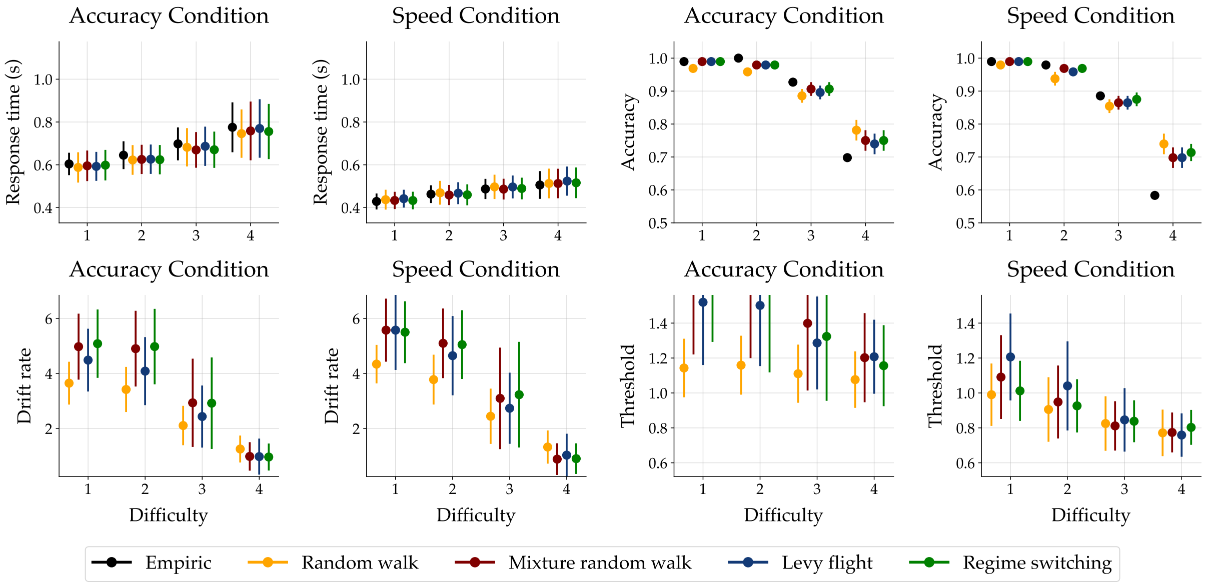

Fig. E9 Aggregate results from all models fitted to the data from participant 4. The top row illustrates posterior re-simulations as a measure of the model's generative performance and absolute goodness-of-fit to the data. The bottom row depicts parameter estimates of the drift rate and the threshold parameter from the non-stationary diffusion decision models (NSDDM). **a** Empirical and re-simulated response times for each difficulty level and both conditions. **b** Empirical and re-simulated proportions of correct choices (accuracy) for each difficulty level and both conditions separately. **c** Posterior estimates of the drift rate parameter for each difficulty level and both conditions separately. **d** Posterior estimates of the threshold parameter for each difficulty level and both conditions separately. Points indicate medians and the error bars represent the median absolute deviations (MAD) across individual data and re-simulations.

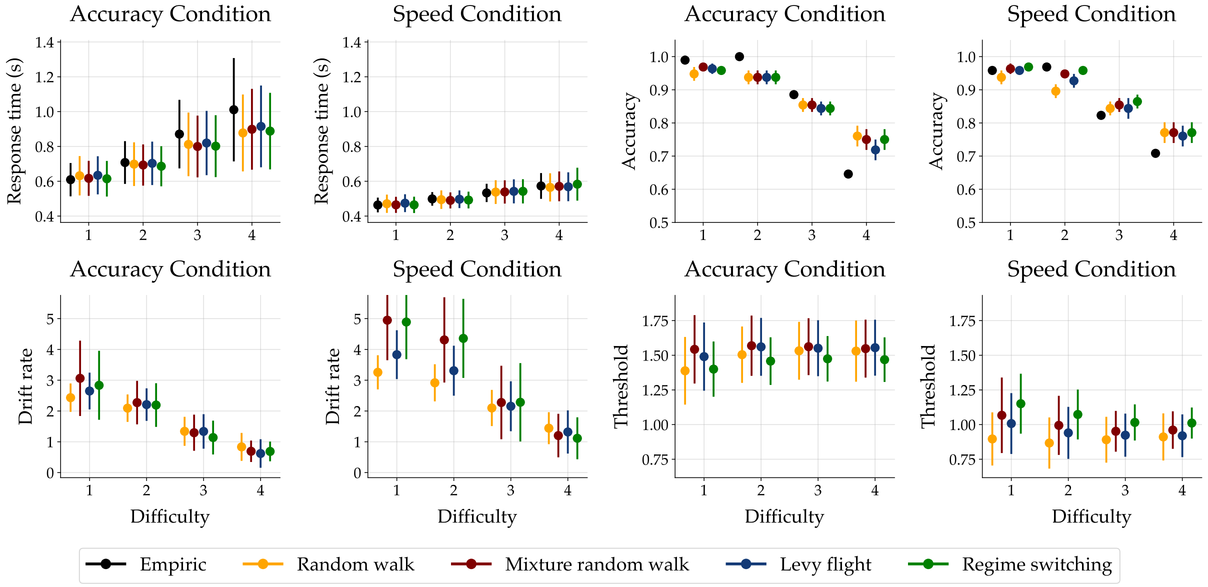

Fig. E10 Aggregate results from all models fitted to the data from participant 5. The top row illustrates posterior re-simulations as a measure of the model's generative performance and absolute goodness-of-fit to the data. The bottom row depicts parameter estimates of the drift rate and the threshold parameter from the non-stationary diffusion decision models (NSDDM). **a** Empirical and re-simulated response times for each difficulty level and both conditions. **b** Empirical and re-simulated proportions of correct choices (accuracy) for each difficulty level and both conditions separately. **c** Posterior estimates of the drift rate parameter for each difficulty level and both conditions separately. **d** Posterior estimates of the threshold parameter for each difficulty level and both conditions separately. Points indicate medians and the error bars represent the median absolute deviations (MAD) across individual data and re-simulations.

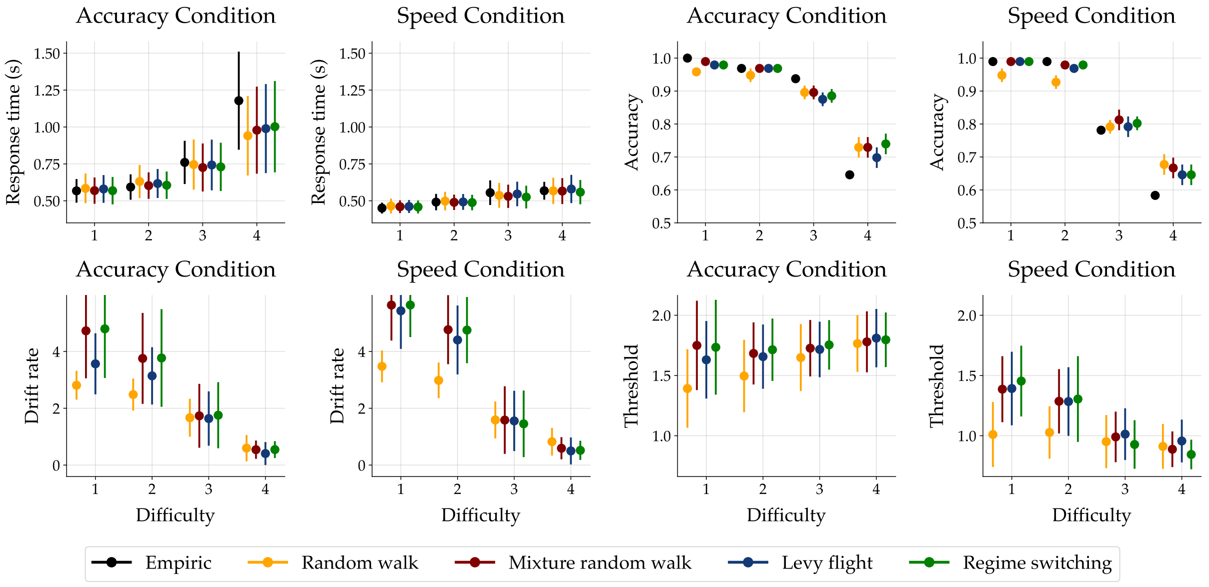

Fig. E11 Aggregate results from all models fitted to the data from participant 6. The top row illustrates posterior re-simulations as a measure of the model's generative performance and absolute goodness-of-fit to the data. The bottom row depicts parameter estimates of the drift rate and the threshold parameter from the non-stationary diffusion decision models (NSDDM). **a** Empirical and re-simulated response times for each difficulty level and both conditions. **b** Empirical and re-simulated proportions of correct choices (accuracy) for each difficulty level and both conditions separately. **c** Posterior estimates of the drift rate parameter for each difficulty level and both conditions separately. **d** Posterior estimates of the threshold parameter for each difficulty level and both conditions separately. Points indicate medians and the error bars represent the median absolute deviations (MAD) across individual data and re-simulations.

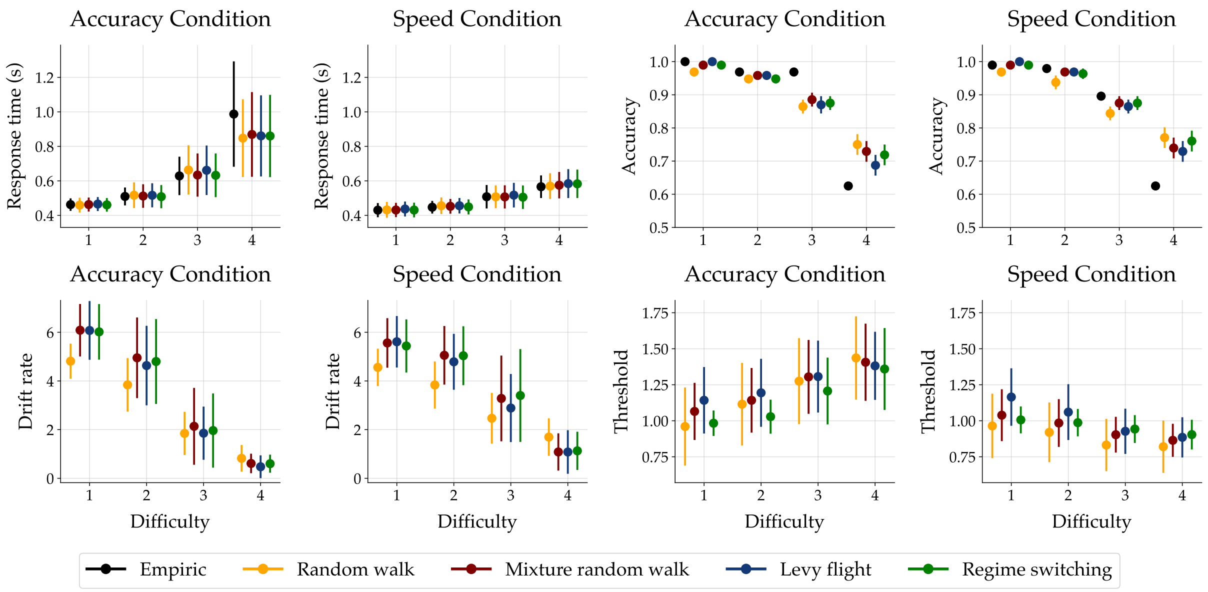

Fig. E12 Aggregate results from all models fitted to the data from participant 7. The top row illustrates posterior re-simulations as a measure of the model's generative performance and absolute goodness-of-fit to the data. The bottom row depicts parameter estimates of the drift rate and the threshold parameter from the non-stationary diffusion decision models (NSDDM). **a** Empirical and re-simulated response times for each difficulty level and both conditions. **b** Empirical and re-simulated proportions of correct choices (accuracy) for each difficulty level and both conditions separately. **c** Posterior estimates of the drift rate parameter for each difficulty level and both conditions separately. **d** Posterior estimates of the threshold parameter for each difficulty level and both conditions separately. Points indicate medians and the error bars represent the median absolute deviations (MAD) across individual data and re-simulations.

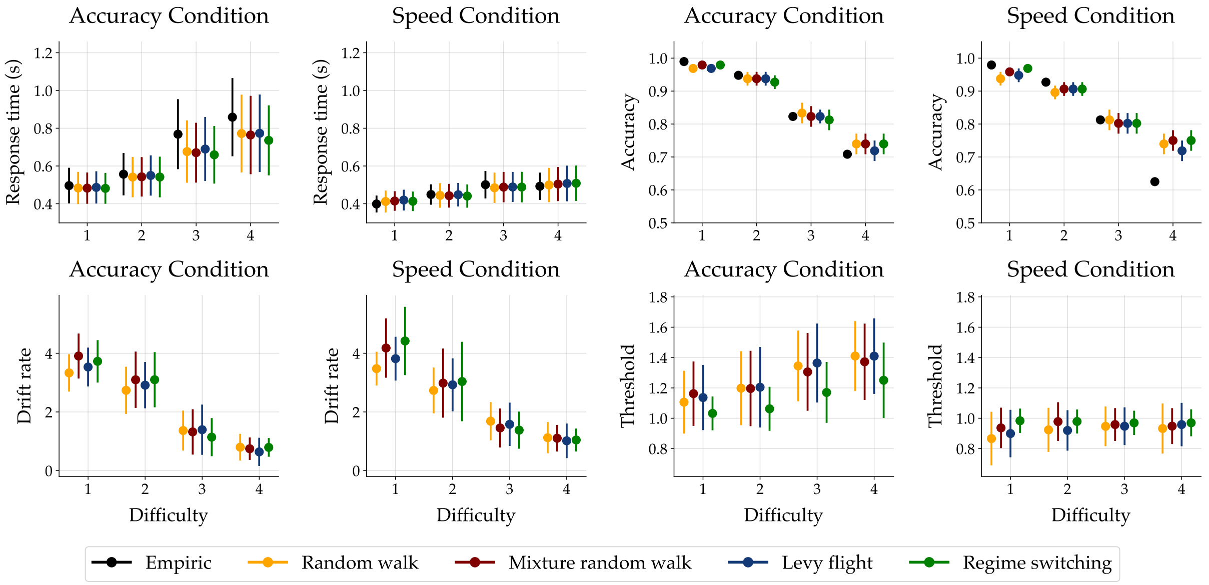

Fig. E13 Aggregate results from all models fitted to the data from participant 8. The top row illustrates posterior re-simulations as a measure of the model's generative performance and absolute goodness-of-fit to the data. The bottom row depicts parameter estimates of the drift rate and the threshold parameter from the non-stationary diffusion decision models (NSDDM). **a** Empirical and re-simulated response times for each difficulty level and both conditions. **b** Empirical and re-simulated proportions of correct choices (accuracy) for each difficulty level and both conditions separately. **c** Posterior estimates of the drift rate parameter for each difficulty level and both conditions separately. **d** Posterior estimates of the threshold parameter for each difficulty level and both conditions separately. Points indicate medians and the error bars represent the median absolute deviations (MAD) across individual data and re-simulations.

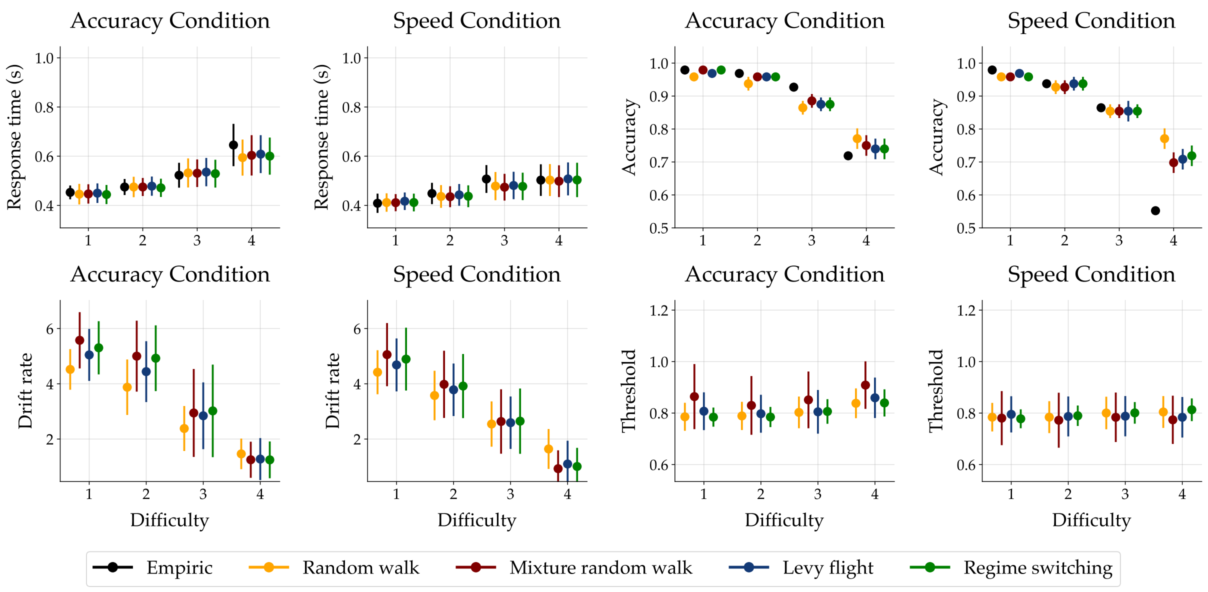

Fig. E14 Aggregate results from all models fitted to the data from participant 9. The top row illustrates posterior re-simulations as a measure of the model's generative performance and absolute goodness-of-fit to the data. The bottom row depicts parameter estimates of the drift rate and the threshold parameter from the non-stationary diffusion decision models (NSDDM). **a** Empirical and re-simulated response times for each difficulty level and both conditions. **b** Empirical and re-simulated proportions of correct choices (accuracy) for each difficulty level and both conditions separately. **c** Posterior estimates of the drift rate parameter for each difficulty level and both conditions separately. **d** Posterior estimates of the threshold parameter for each difficulty level and both conditions separately. Points indicate medians and the error bars represent the median absolute deviations (MAD) across individual data and re-simulations.

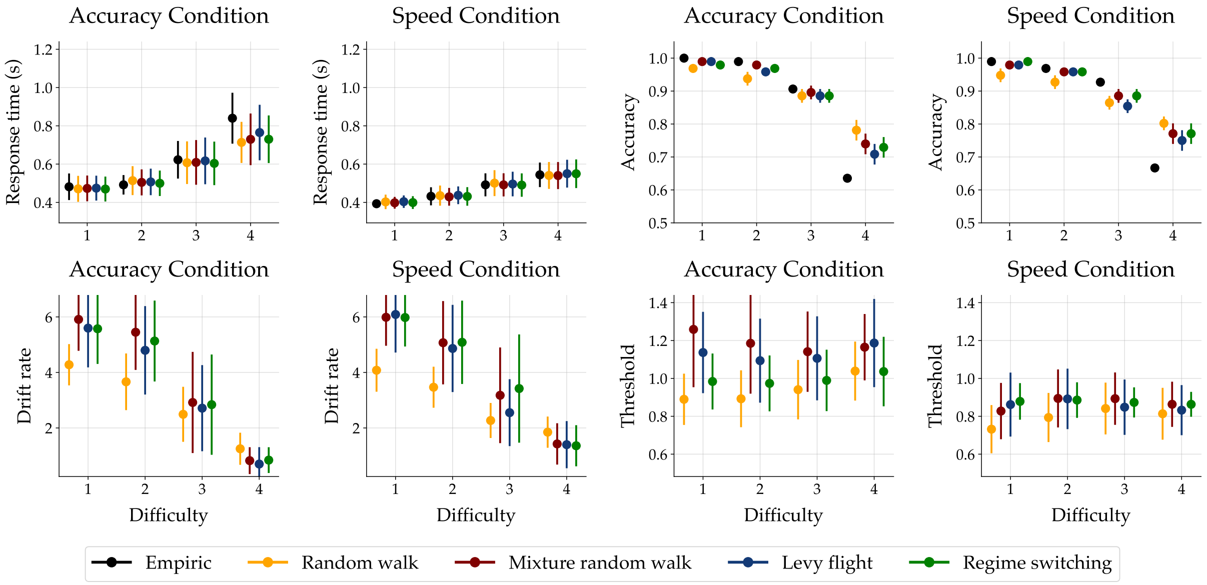

Fig. E15 Aggregate results from all models fitted to the data from participant 10. The top row illustrates posterior re-simulations as a measure of the model's generative performance and absolute goodness-of-fit to the data. The bottom row depicts parameter estimates of the drift rate and the threshold parameter from the non-stationary diffusion decision models (NSDDM). **a** Empirical and re-simulated response times for each difficulty level and both conditions. **b** Empirical and re-simulated proportions of correct choices (accuracy) for each difficulty level and both conditions separately. **c** Posterior estimates of the drift rate parameter for each difficulty level and both conditions separately. **d** Posterior estimates of the threshold parameter for each difficulty level and both conditions separately. Points indicate medians and the error bars represent the median absolute deviations (MAD) across individual data and re-simulations.

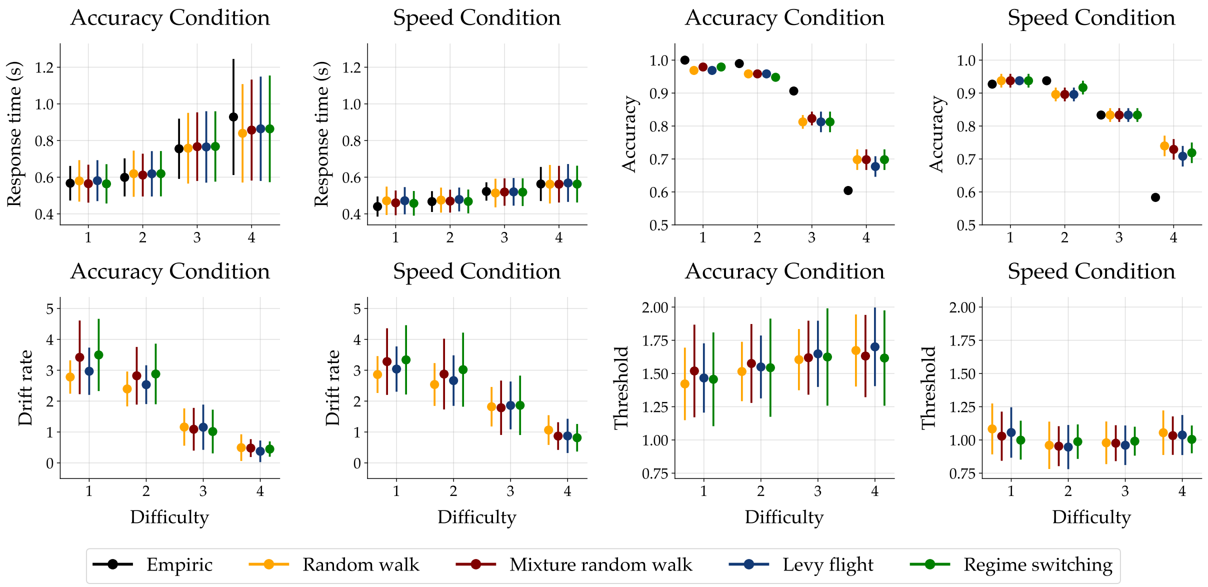

Fig. E16 Aggregate results from all models fitted to the data from participant 11. The top row illustrates posterior re-simulations as a measure of the model's generative performance and absolute goodness-of-fit to the data. The bottom row depicts parameter estimates of the drift rate and the threshold parameter from the non-stationary diffusion decision models (NSDDM). **a** Empirical and re-simulated response times for each difficulty level and both conditions. **b** Empirical and re-simulated proportions of correct choices (accuracy) for each difficulty level and both conditions separately. **c** Posterior estimates of the drift rate parameter for each difficulty level and both conditions separately. **d** Posterior estimates of the threshold parameter for each difficulty level and both conditions separately. Points indicate medians and the error bars represent the median absolute deviations (MAD) across individual data and re-simulations.

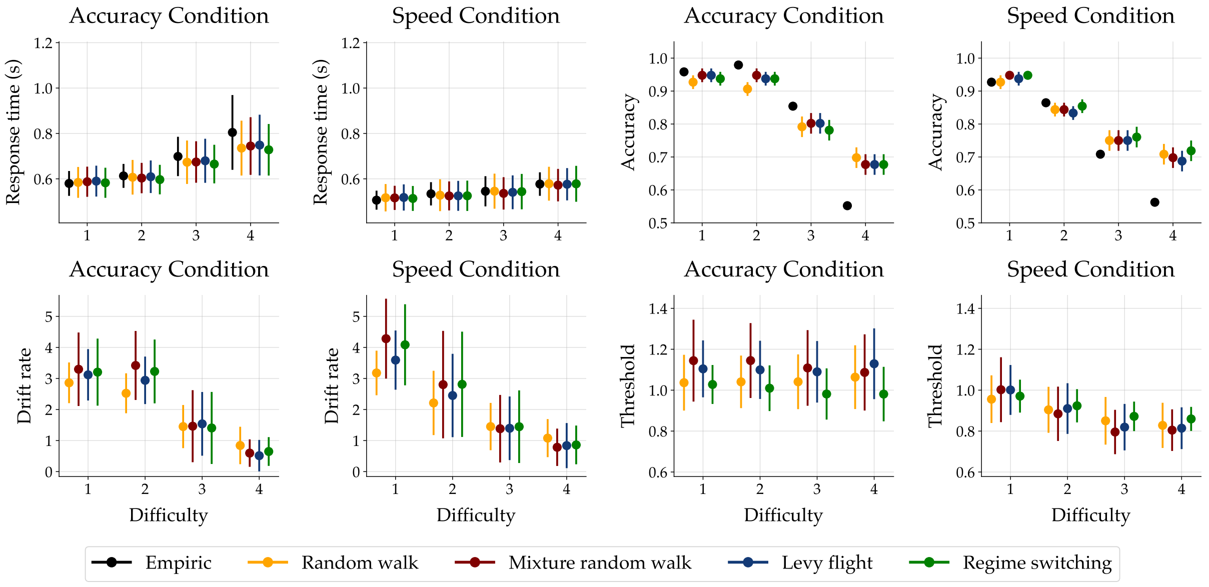

Fig. E17 Aggregate results from all models fitted to the data from participant 12. The top row illustrates posterior re-simulations as a measure of the model's generative performance and absolute goodness-of-fit to the data. The bottom row depicts parameter estimates of the drift rate and the threshold parameter from the non-stationary diffusion decision models (NSDDM). **a** Empirical and re-simulated response times for each difficulty level and both conditions. **b** Empirical and re-simulated proportions of correct choices (accuracy) for each difficulty level and both conditions separately. **c** Posterior estimates of the drift rate parameter for each difficulty level and both conditions separately. **d** Posterior estimates of the threshold parameter for each difficulty level and both conditions separately. Points indicate medians and the error bars represent the median absolute deviations (MAD) across individual data and re-simulations.

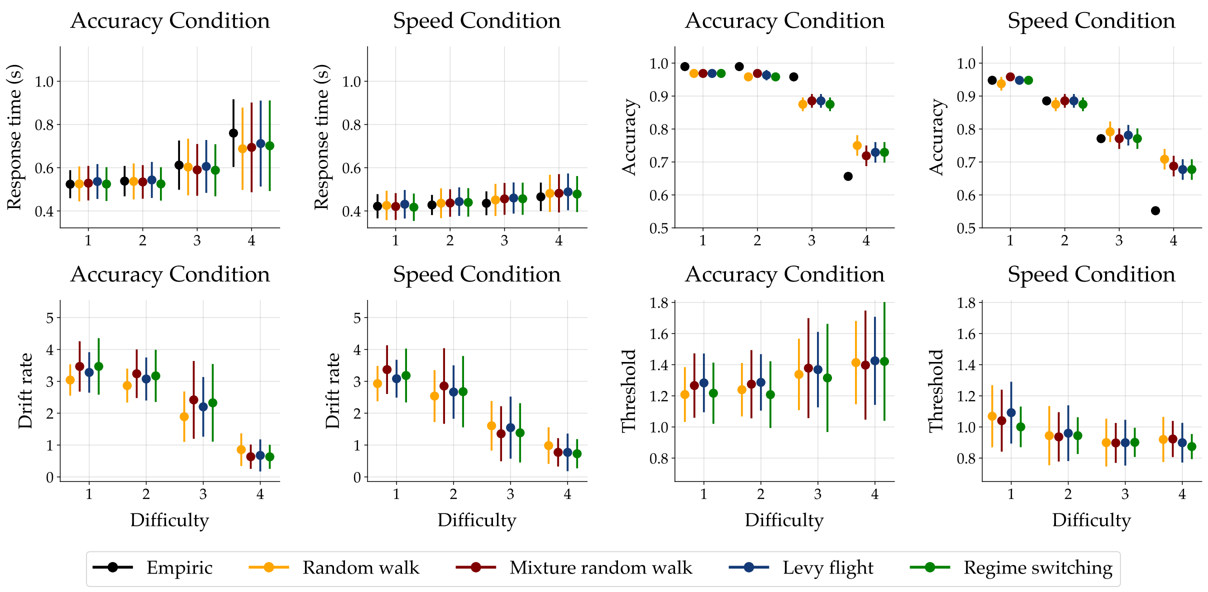

Fig. E18 Aggregate results from all models fitted to the data from participant 13. The top row illustrates posterior re-simulations as a measure of the model's generative performance and absolute goodness-of-fit to the data. The bottom row depicts parameter estimates of the drift rate and the threshold parameter from the non-stationary diffusion decision models (NSDDM). **a** Empirical and re-simulated response times for each difficulty level and both conditions. **b** Empirical and re-simulated proportions of correct choices (accuracy) for each difficulty level and both conditions separately. **c** Posterior estimates of the drift rate parameter for each difficulty level and both conditions separately. **d** Posterior estimates of the threshold parameter for each difficulty level and both conditions separately. Points indicate medians and the error bars represent the median absolute deviations (MAD) across individual data and re-simulations.

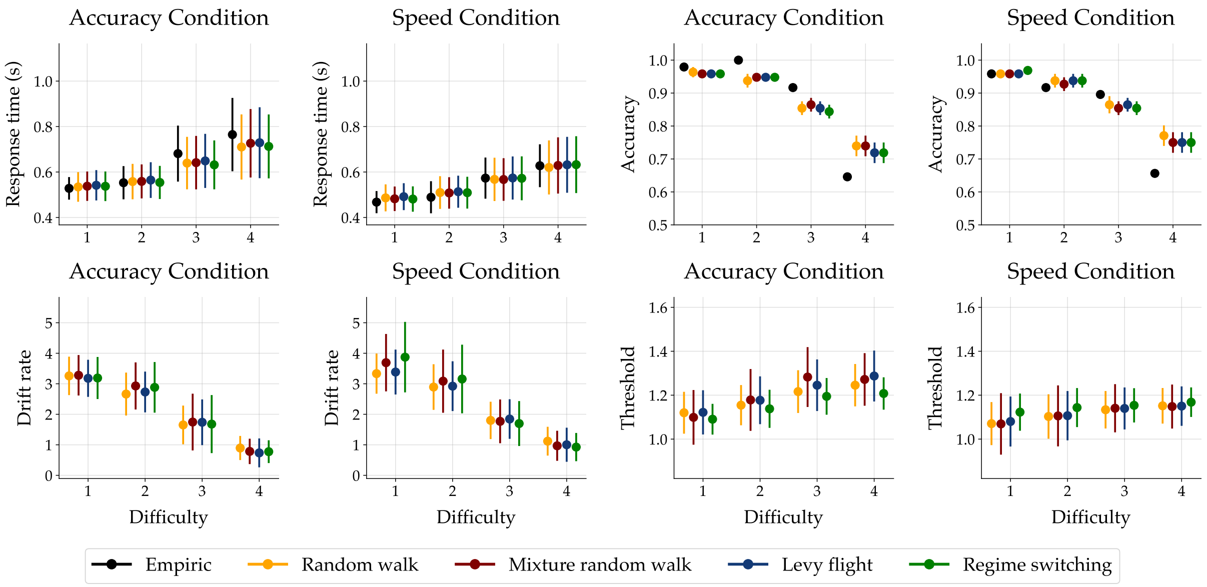

Fig. E19 Aggregate results from all models fitted to the data from participant 14. The top row illustrates posterior re-simulations as a measure of the model's generative performance and absolute goodness-of-fit to the data. The bottom row depicts parameter estimates of the drift rate and the threshold parameter from the non-stationary diffusion decision models (NSDDM). **a** Empirical and re-simulated response times for each difficulty level and both conditions. **b** Empirical and re-simulated proportions of correct choices (accuracy) for each difficulty level and both conditions separately. **c** Posterior estimates of the drift rate parameter for each difficulty level and both conditions separately. **d** Posterior estimates of the threshold parameter for each difficulty level and both conditions separately. Points indicate medians and the error bars represent the median absolute deviations (MAD) across individual data and re-simulations.

Appendix F Response Time Time Series

In the following, we present the model fit to the whole response time time series for the remaining 12 participants.

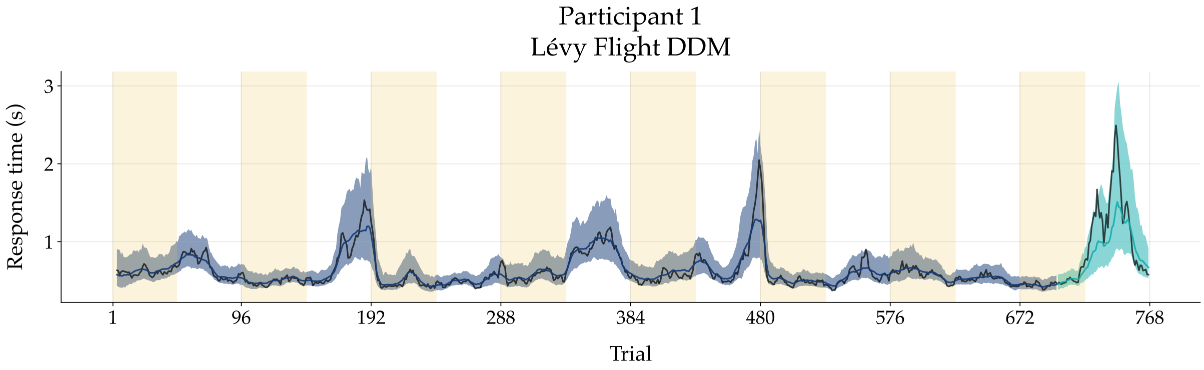

Fig. F20 Model fit to response time (RT) time series. The empirical RT time series of participant 1 is shown in black. From trial 1 to 700, the posterior re-simulation (aka retrodictive check) using the best fitting non-stationary diffusion decision model (NSDDM) for this specific individual are shown in blue. In this instance, the results stem from a Lévy flight DDM. For the remaining trials, one-step-ahead predictions are depicted in cyan. Solid lines correspond to the median and shaded bands to 90% credibility intervals (CI). The empirical, re-simulated, and predicted RT time series were smoothed via a simple moving average (SMA) with a period of 5. The yellow shaded regions indicate trials where speed was emphasised over accuracy, while blank white areas denote instances where the opposite emphasis was applied.

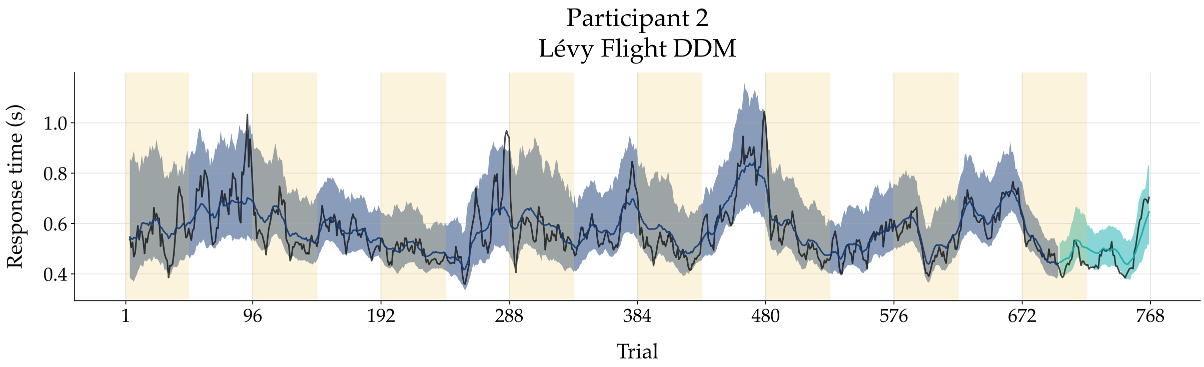

Fig. F21 Model fit to response time (RT) time series. The empirical RT time series of participant 2 is shown in black. From trial 1 to 700, the posterior re-simulation (aka retrodictive check) using the best fitting non-stationary diffusion decision model (NSDDM) for this specific individual are shown in blue. In this instance, the results stem from a Lévy flight DDM. For the remaining trials, one-step-ahead predictions are depicted in cyan. Solid lines correspond to the median and shaded bands to 90% credibility intervals (CI). The empirical, re-simulated, and predicted RT time series were smoothed via a simple moving average (SMA) with a period of 5. The yellow shaded regions indicate trials where speed was emphasised over accuracy, while blank white areas denote instances where the opposite emphasis was applied.

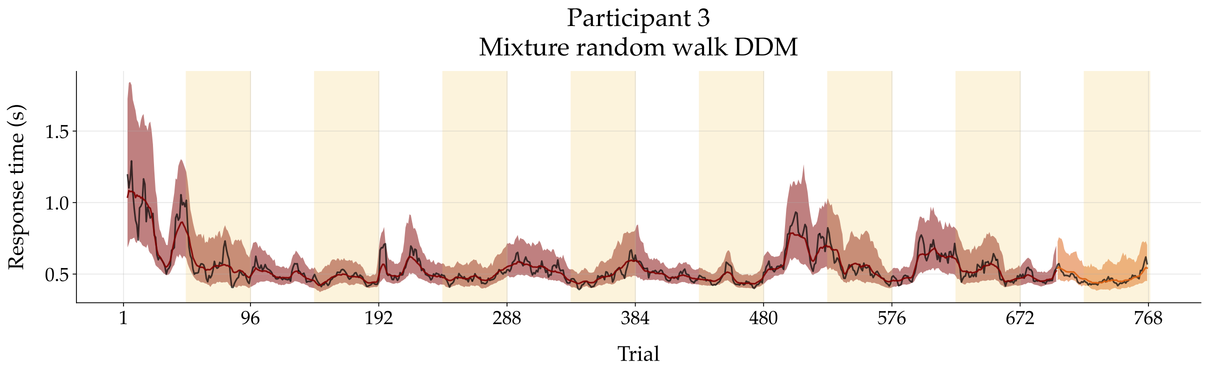

Fig. F22 Model fit to response time (RT) time series. The empirical RT time series of participant 3 is shown in black. From trial 1 to 700, the posterior re-simulation (aka retrodictive check) using the best fitting non-stationary diffusion decision model (NSDDM) for this specific individual are shown in red. In this instance, the results stem from a mixture random walk DDM. For the remaining trials, one-step-ahead predictions are depicted in orange. Solid lines correspond to the median and shaded bands to 90% credibility intervals (CI). The empirical, re-simulated, and predicted RT time series were smoothed via a simple moving average (SMA) with a period of 5. The yellow shaded regions indicate trials where speed was emphasised over accuracy, while blank white areas denote instances where the opposite emphasis was applied.

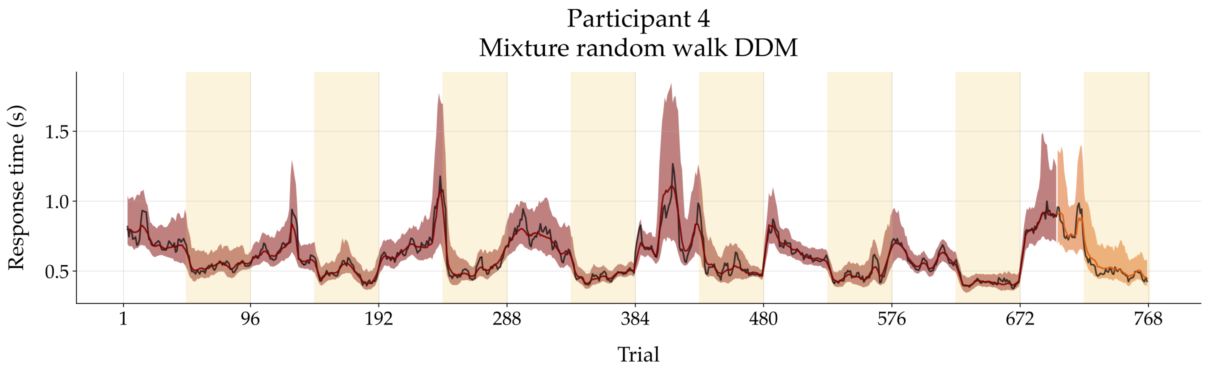

Fig. F23 Model fit to response time (RT) time series. The empirical RT time series of participant 4 is shown in black. From trial 1 to 700, the posterior re-simulation (aka retrodictive check) using the best fitting non-stationary diffusion decision model (NSDDM) for this specific individual are shown in red. In this instance, the results stem from a mixture random walk DDM. For the remaining trials, one-step-ahead predictions are depicted in orange. Solid lines correspond to the median and shaded bands to 90% credibility intervals (CI). The empirical, re-simulated, and predicted RT time series were smoothed via a simple moving average (SMA) with a period of 5. The yellow shaded regions indicate trials where speed was emphasised over accuracy, while blank white areas denote instances where the opposite emphasis was applied.

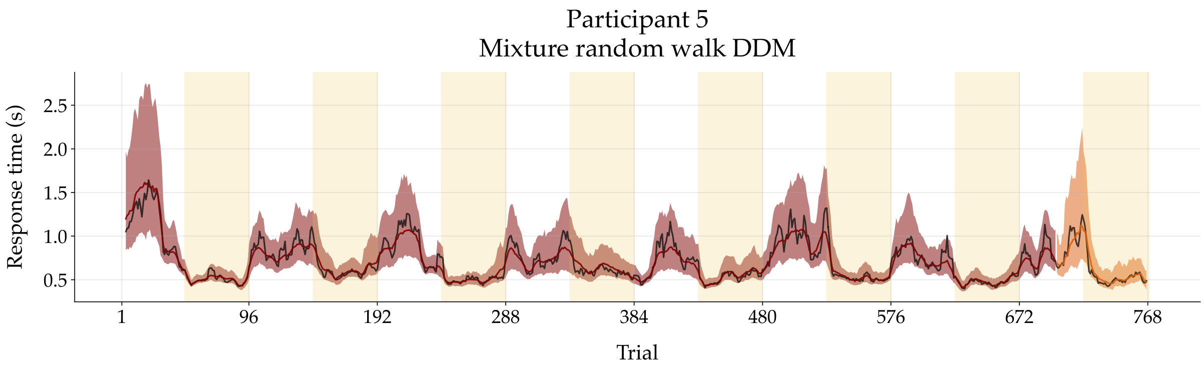

Fig. F24 Model fit to response time (RT) time series. The empirical RT time series of participant 5 is shown in black. From trial 1 to 700, the posterior re-simulation (aka retrodictive check) using the best fitting non-stationary diffusion decision model (NSDDM) for this specific individual are shown in red. In this instance, the results stem from a mixture random walk DDM. For the remaining trials, one-step-ahead predictions are depicted in orange. Solid lines correspond to the median and shaded bands to 90% credibility intervals (CI). The empirical, re-simulated, and predicted RT time series were smoothed via a simple moving average (SMA) with a period of 5. The yellow shaded regions indicate trials where speed was emphasised over accuracy, while blank white areas denote instances where the opposite emphasis was applied.

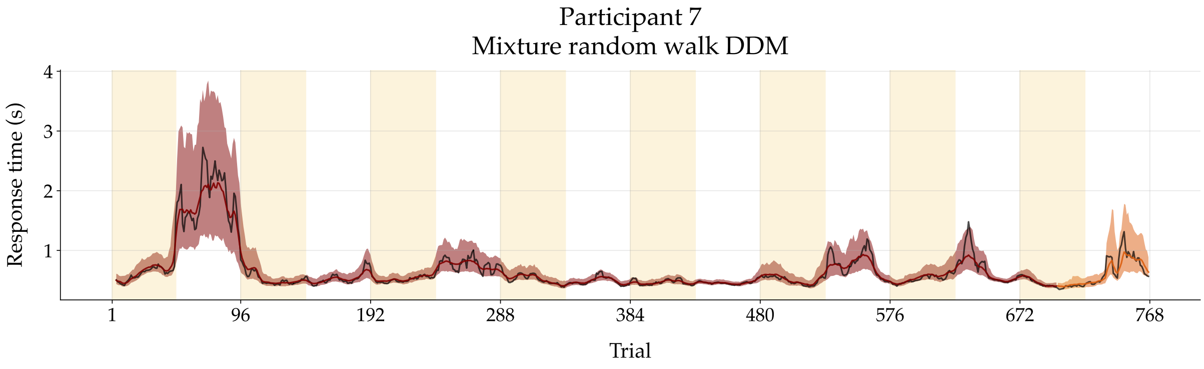

Fig. F25 Model fit to response time (RT) time series. The empirical RT time series of participant 7 is shown in black. From trial 1 to 700, the posterior re-simulation (aka retrodictive check) using the best fitting non-stationary diffusion decision model (NSDDM) for this specific individual are shown in red. In this instance, the results stem from a mixture random walk DDM. For the remaining trials, one-step-ahead predictions are depicted in orange. Solid lines correspond to the median and shaded bands to 90% credibility intervals (CI). The empirical, re-simulated, and predicted RT time series were smoothed via a simple moving average (SMA) with a period of 5. The yellow shaded regions indicate trials where speed was emphasised over accuracy, while blank white areas denote instances where the opposite emphasis was applied.

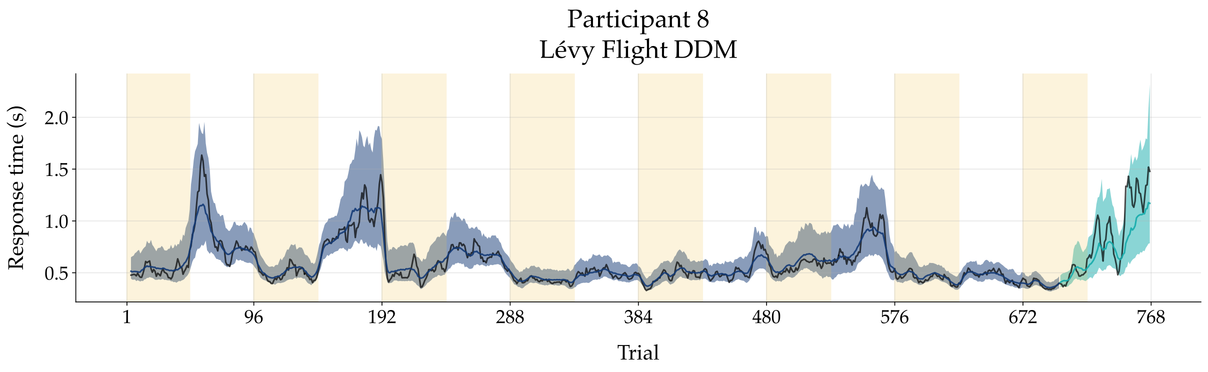

Fig. F26 Model fit to response time (RT) time series. The empirical RT time series of participant 8 is shown in black. From trial 1 to 700, the posterior re-simulation (aka retrodictive check) using the best fitting non-stationary diffusion decision model (NSDDM) for this specific individual are shown in blue. In this instance, the results stem from a Lévy flight DDM. For the remaining trials, one-step-ahead predictions are depicted in cyan. Solid lines correspond to the median and shaded bands to 90% credibility intervals (CI). The empirical, re-simulated, and predicted RT time series were smoothed via a simple moving average (SMA) with a period of 5. The yellow shaded regions indicate trials where speed was emphasised over accuracy, while blank white areas denote instances where the opposite emphasis was applied.

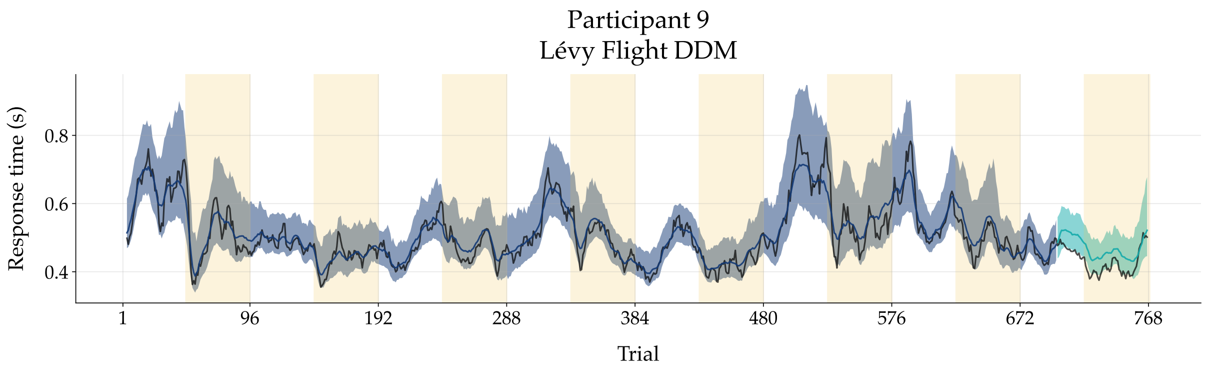

Fig. F27 Model fit to response time (RT) time series. The empirical RT time series of participant 9 is shown in black. From trial 1 to 700, the posterior re-simulation (aka retrodictive check) using the best fitting non-stationary diffusion decision model (NSDDM) for this specific individual are shown in blue. In this instance, the results stem from a Lévy flight DDM. For the remaining trials, one-step-ahead predictions are depicted in cyan. Solid lines correspond to the median and shaded bands to 90% credibility intervals (CI). The empirical, re-simulated, and predicted RT time series were smoothed via a simple moving average (SMA) with a period of 5. The yellow shaded regions indicate trials where speed was emphasised over accuracy, while blank white areas denote instances where the opposite emphasis was applied.

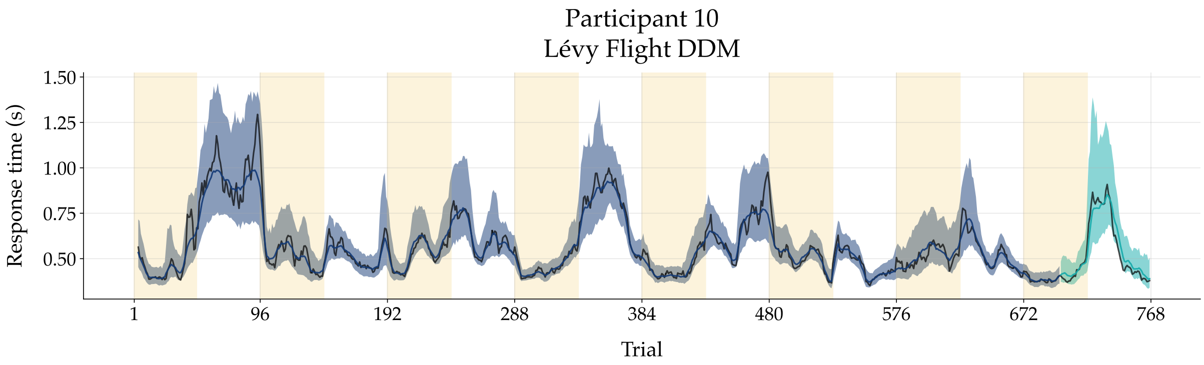

Fig. F28 Model fit to response time (RT) time series. The empirical RT time series of participant 10 is shown in black. From trial 1 to 700, the posterior re-simulation (aka retrodictive check) using the best fitting non-stationary diffusion decision model (NSDDM) for this specific individual are shown in blue. In this instance, the results stem from a Lévy flight DDM. For the remaining trials, one-step-ahead predictions are depicted in cyan. Solid lines correspond to the median and shaded bands to 90% credibility intervals (CI). The empirical, re-simulated, and predicted RT time series were smoothed via a simple moving average (SMA) with a period of 5. The yellow shaded regions indicate trials where speed was emphasised over accuracy, while blank white areas denote instances where the opposite emphasis was applied.

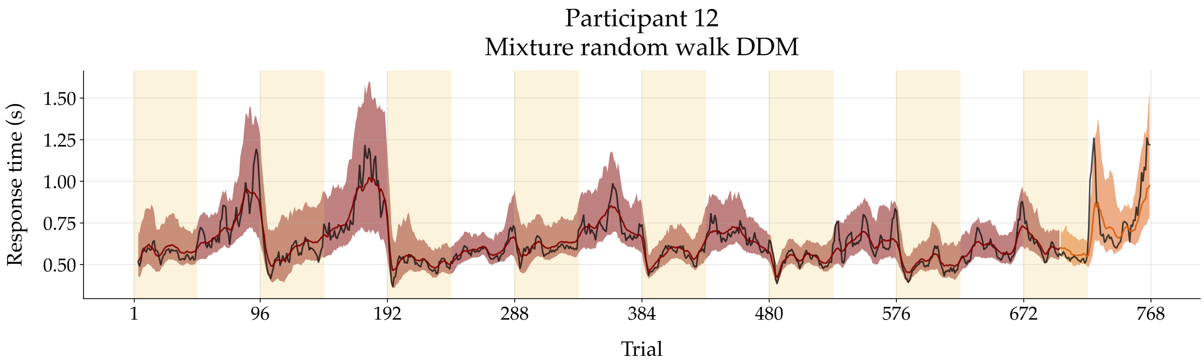

Fig. F29 Model fit to response time (RT) time series. The empirical RT time series of participant 7 is shown in black. From trial 1 to 700, the posterior re-simulation (aka retrodictive check) using the best fitting non-stationary diffusion decision model (NSDDM) for this specific individual are shown in red. In this instance, the results stem from a mixture random walk DDM. For the remaining trials, one-step-ahead predictions are depicted in orange. Solid lines correspond to the median and shaded bands to 90% credibility intervals (CI). The empirical, re-simulated, and predicted RT time series were smoothed via a simple moving average (SMA) with a period of 5. The yellow shaded regions indicate trials where speed was emphasised over accuracy, while blank white areas denote instances where the opposite emphasis was applied.

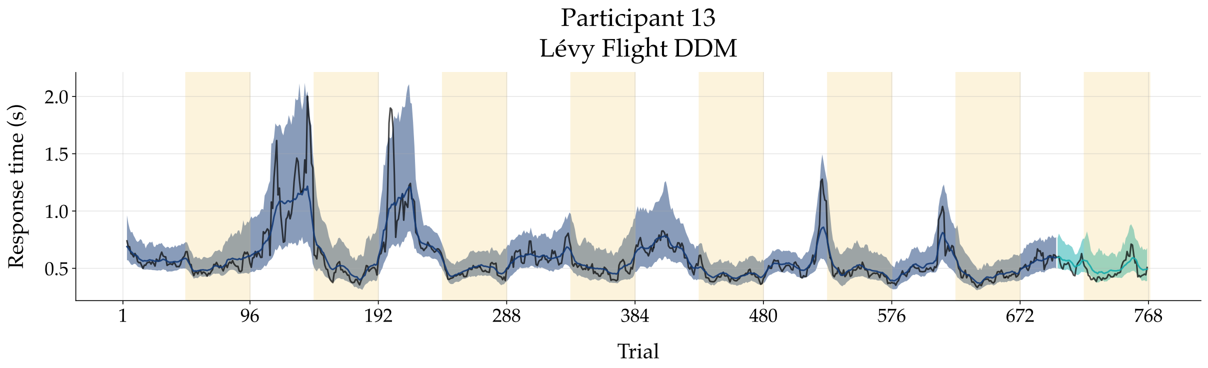

Fig. F30 Model fit to response time (RT) time series. The empirical RT time series of participant 13 is shown in black. From trial 1 to 700, the posterior re-simulation (aka retrodictive check) using the best fitting non-stationary diffusion decision model (NSDDM) for this specific individual are shown in blue. In this instance, the results stem from a Lévy flight DDM. For the remaining trials, one-step-ahead predictions are depicted in cyan. Solid lines correspond to the median and shaded bands to 90% credibility intervals (CI). The empirical, re-simulated, and predicted RT time series were smoothed via a simple moving average (SMA) with a period of 5. The yellow shaded regions indicate trials where speed was emphasised over accuracy, while blank white areas denote instances where the opposite emphasis was applied.

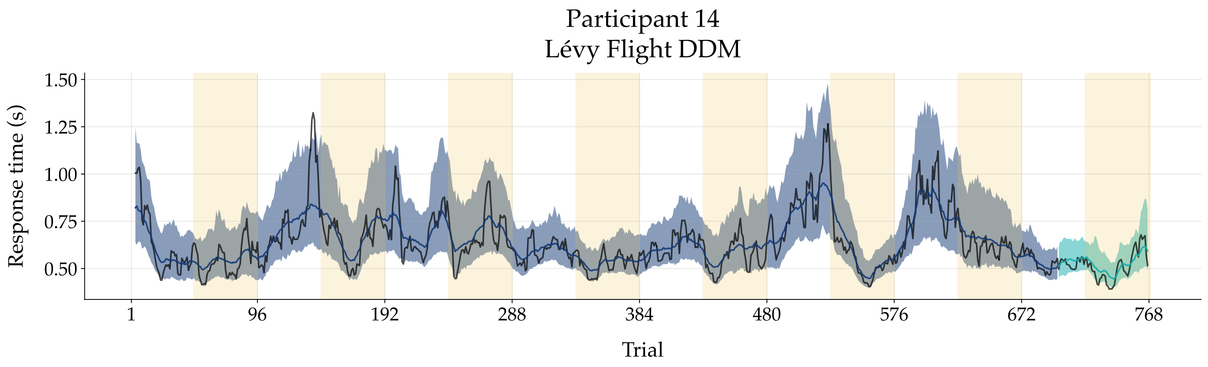

Fig. F31 Model fit to response time (RT) time series. The empirical RT time series of participant 14 is shown in black. From trial 1 to 700, the posterior re-simulation (aka retrodictive check) using the best fitting non-stationary diffusion decision model (NSDDM) for this specific individual are shown in blue. In this instance, the results stem from a Lévy flight DDM. For the remaining trials, one-step-ahead predictions are depicted in cyan. Solid lines correspond to the median and shaded bands to 90% credibility intervals (CI). The empirical, re-simulated, and predicted RT time series were smoothed via a simple moving average (SMA) with a period of 5. The yellow shaded regions indicate trials where speed was emphasised over accuracy, while blank white areas denote instances where the opposite emphasis was applied.

Appendix G Parameter Trajectories

In the following, we present the inferred parameter trajectories for the remaining participants. For each visualisation the model with the highest posterior model probability for that specific individual was used.

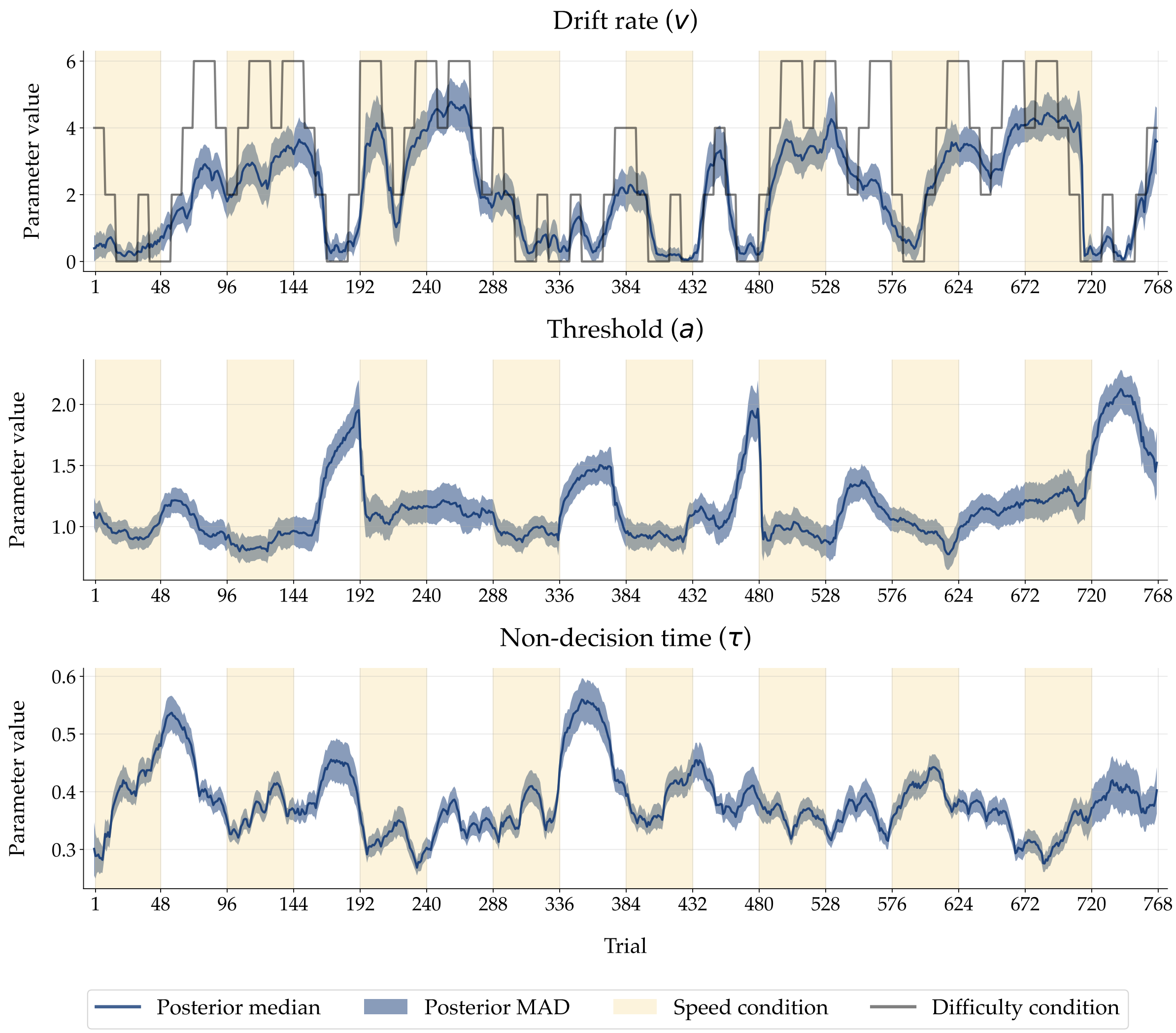

Fig. G32 Posterior parameter trajectory inferred with the best fitting NSDDM of participant 1 (a Lévy flight DDM in this case) for all three DDM parameters (drift rate, threshold, and non-decision time) separately. The yellow shaded areas indicate trials where speed was emphasised over accuracy and blank white area indicated where the opposite was asked for. In the top panel, the task difficulty levels sequence is depicted in black lines.

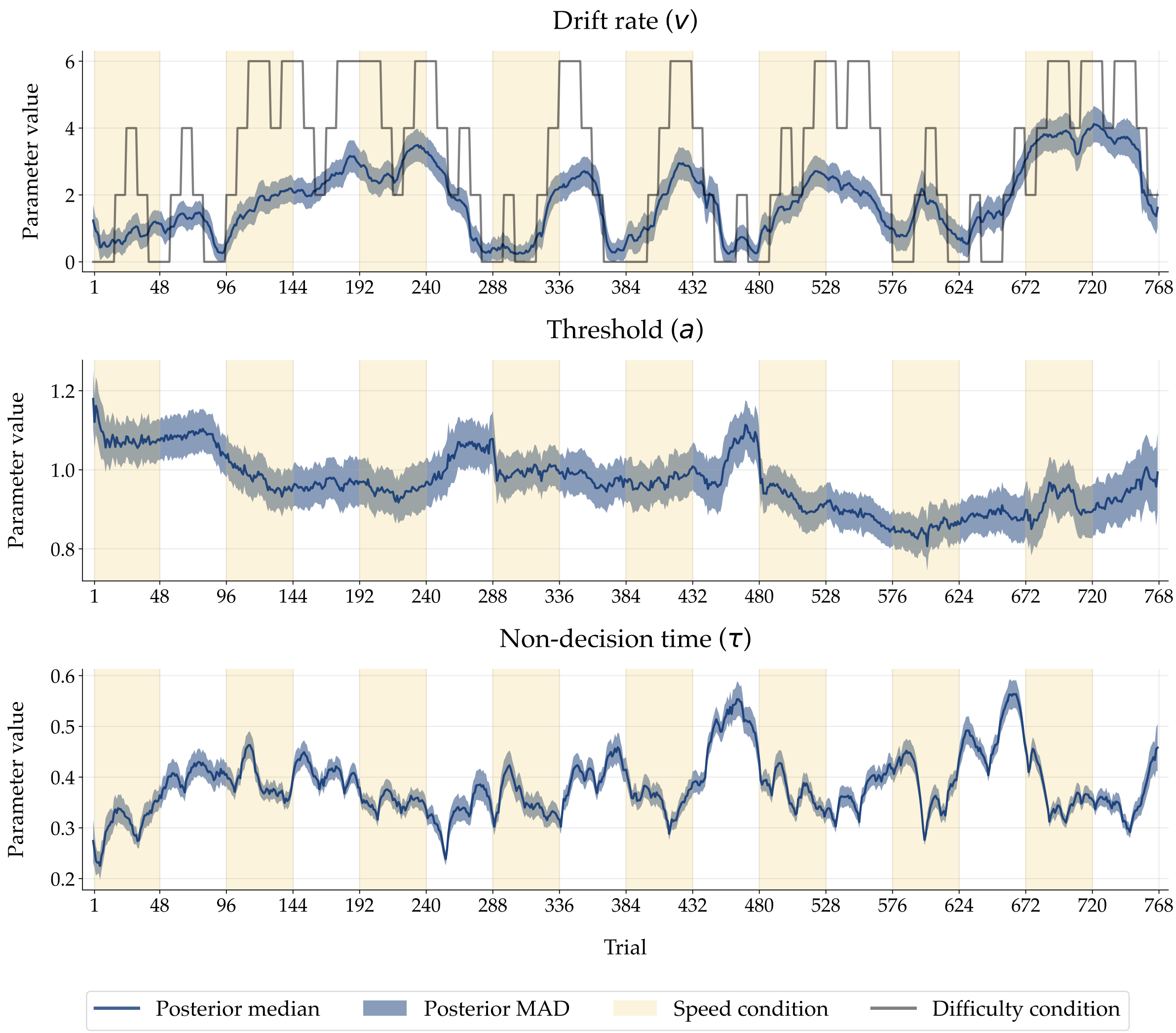

Fig. G33 Posterior parameter trajectory inferred with the best fitting NSDDM of participant 2 (a Lévy flight DDM in this case) for all three DDM parameters (drift rate, threshold, and non-decision time) separately. The yellow shaded areas indicate trials where speed was emphasised over accuracy and blank white area indicated where the opposite was asked for. In the top panel, the task difficulty levels sequence is depicted in black lines.

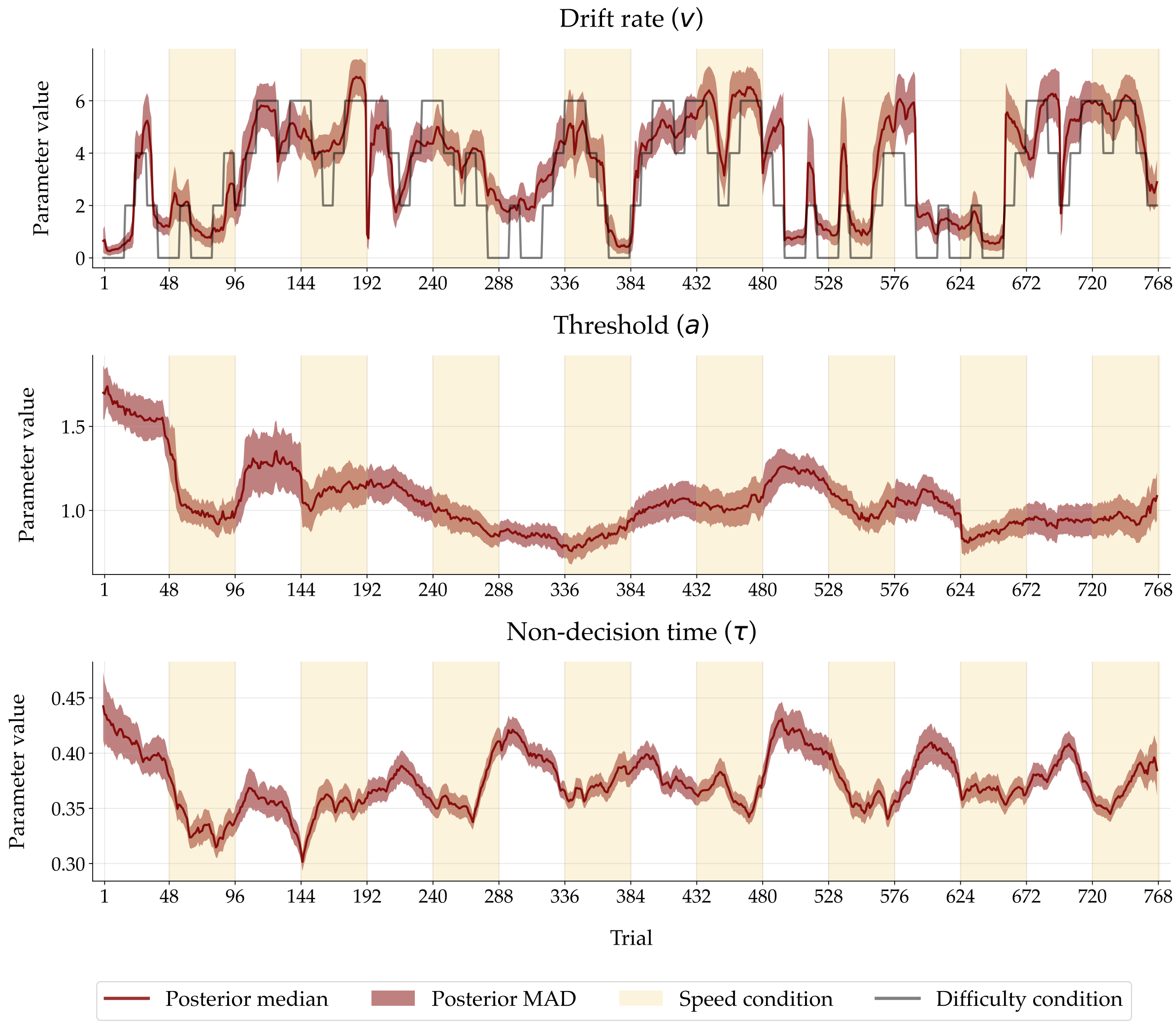

Fig. G34 Posterior parameter trajectory inferred with the best fitting NSDDM of participant 3 (a mixture random walk DDM in this case) for all three DDM parameters (drift rate, threshold, and non-decision time) separately. The yellow shaded areas indicate trials where speed was emphasised over accuracy and blank white area indicated where the opposite was asked for. In the top panel, the task difficulty levels sequence is depicted in black lines.

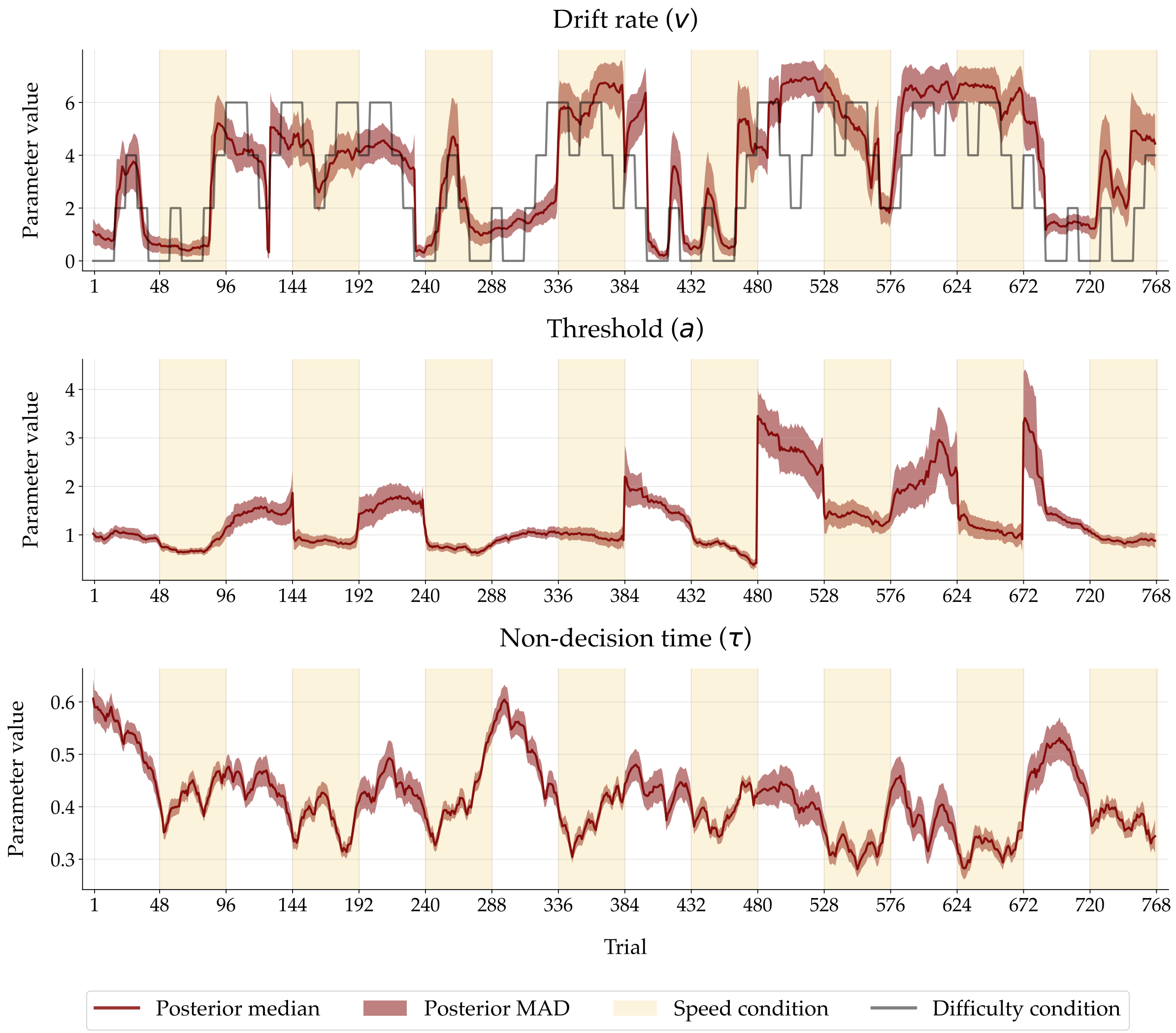

Fig. G35 Posterior parameter trajectory inferred with the best fitting NSDDM of participant 4 (a mixture random walk DDM in this case) for all three DDM parameters (drift rate, threshold, and non-decision time) separately. The yellow shaded areas indicate trials where speed was emphasised over accuracy and blank white area indicated where the opposite was asked for. In the top panel, the task difficulty levels sequence is depicted in black lines.

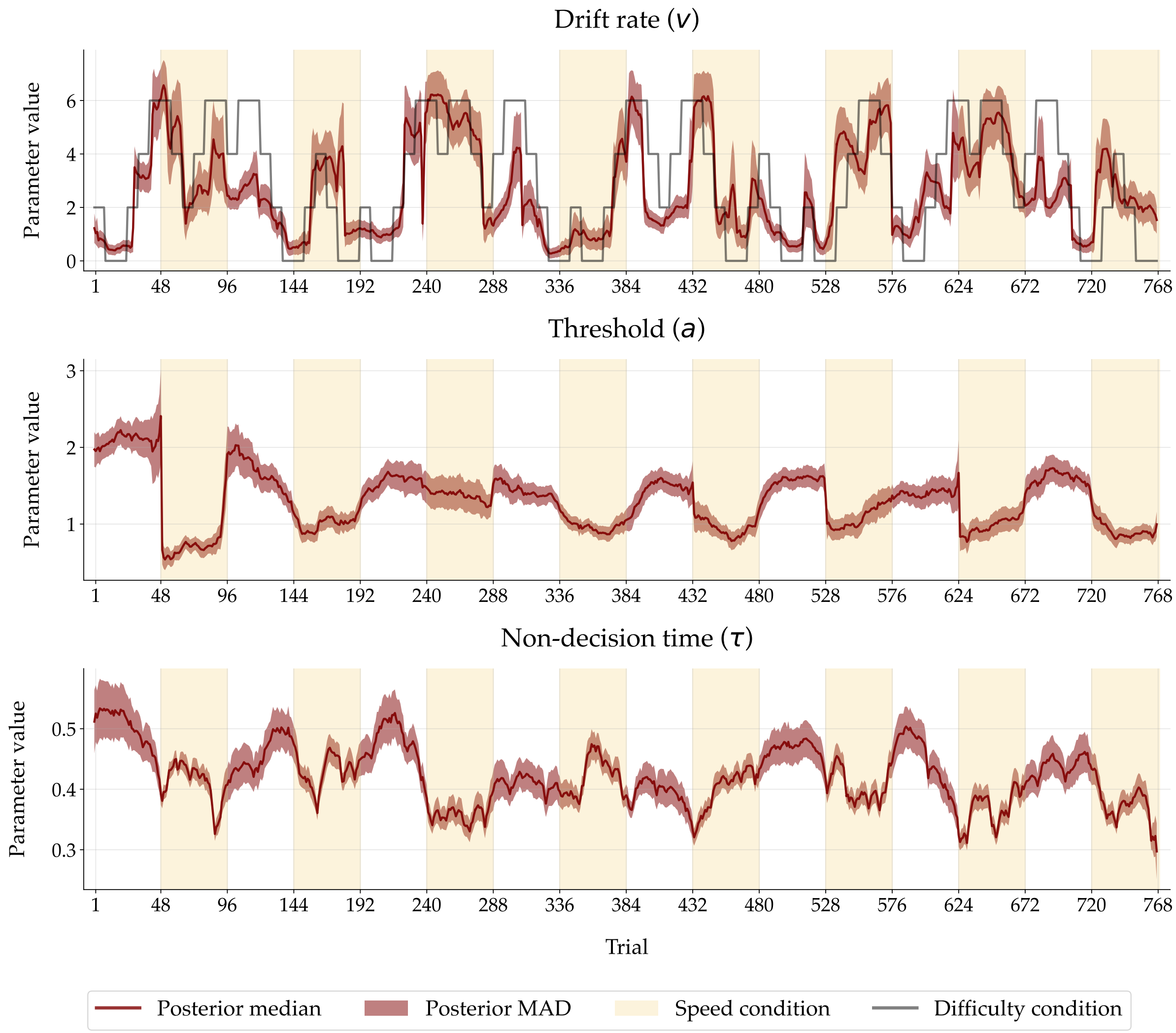

Fig. G36 Posterior parameter trajectory inferred with the best fitting NSDDM of participant 5 (a mixture random walk DDM in this case) for all three DDM parameters (drift rate, threshold, and non-decision time) separately. The yellow shaded areas indicate trials where speed was emphasised over accuracy and blank white area indicated where the opposite was asked for. In the top panel, the task difficulty levels sequence is depicted in black lines.

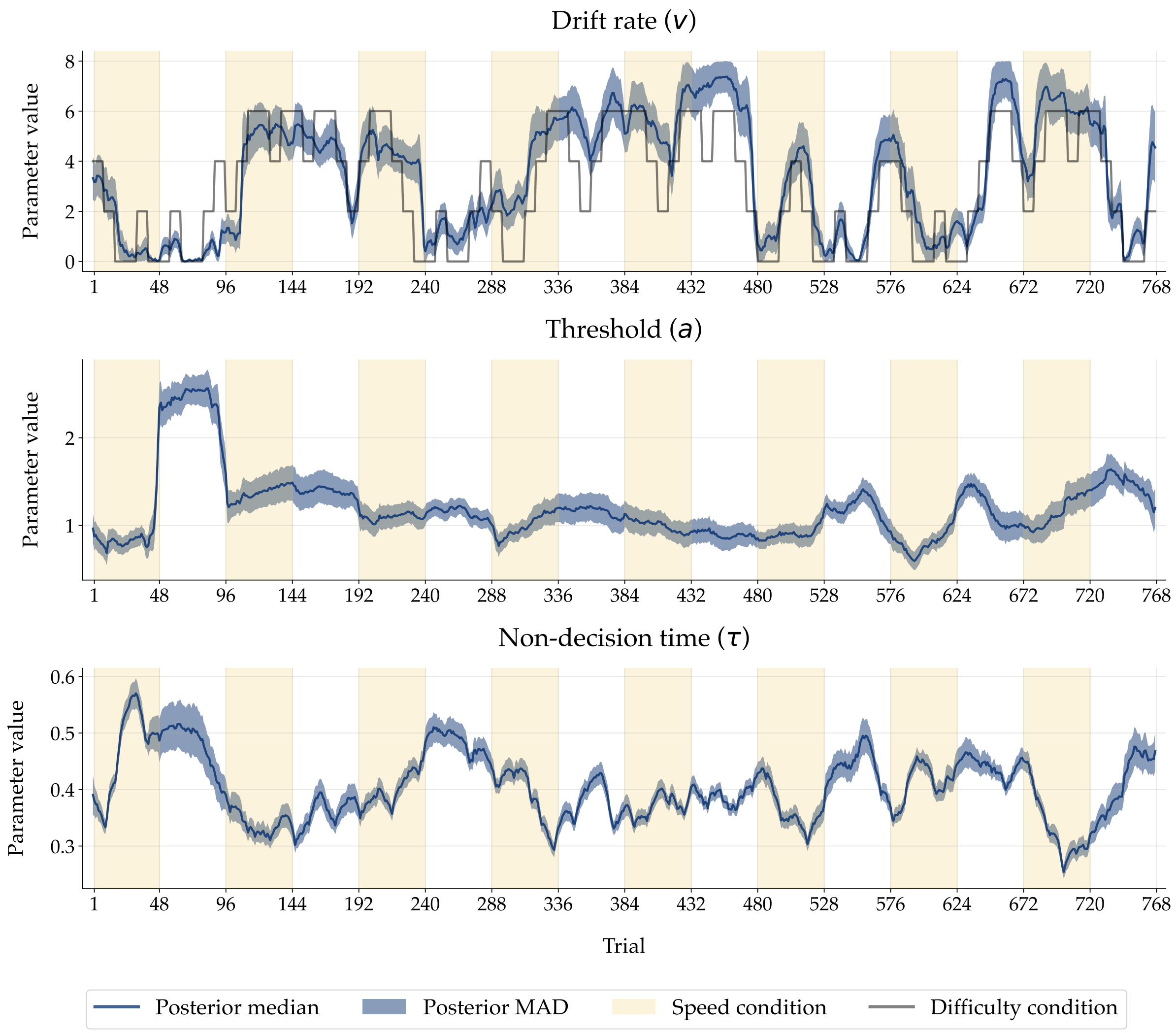

Fig. G37 Posterior parameter trajectory inferred with the best fitting NSDDM of participant 7 (a Lévy flight DDM in this case) for all three DDM parameters (drift rate, threshold, and non-decision time) separately. The yellow shaded areas indicate trials where speed was emphasised over accuracy and blank white area indicated where the opposite was asked for. In the top panel, the task difficulty levels sequence is depicted in black lines.

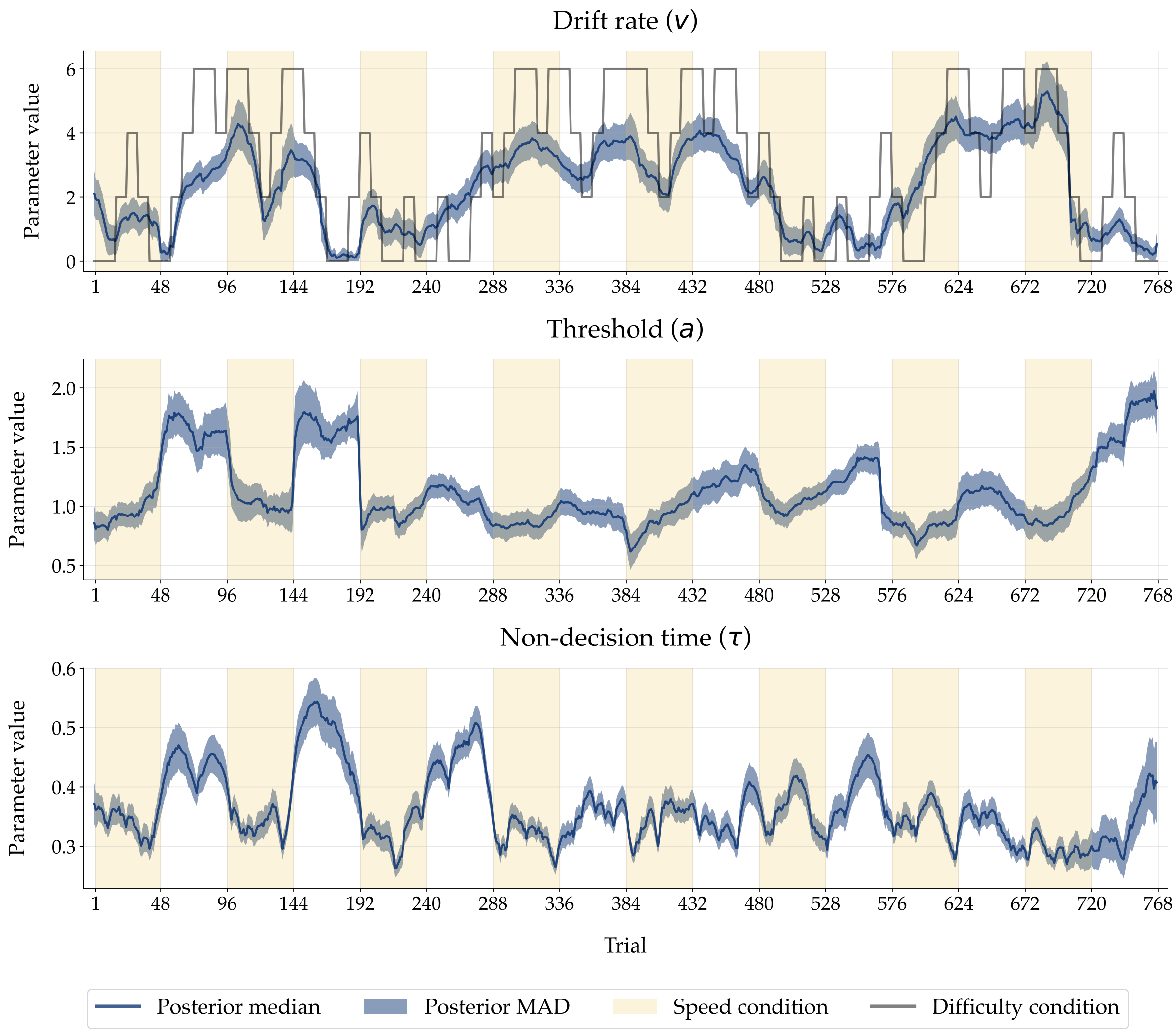

Fig. G38 Posterior parameter trajectory inferred with the best fitting NSDDM of participant 8 (a Lévy flight DDM in this case) for all three DDM parameters (drift rate, threshold, and non-decision time) separately. The yellow shaded areas indicate trials where speed was emphasised over accuracy and blank white area indicated where the opposite was asked for. In the top panel, the task difficulty levels sequence is depicted in black lines.

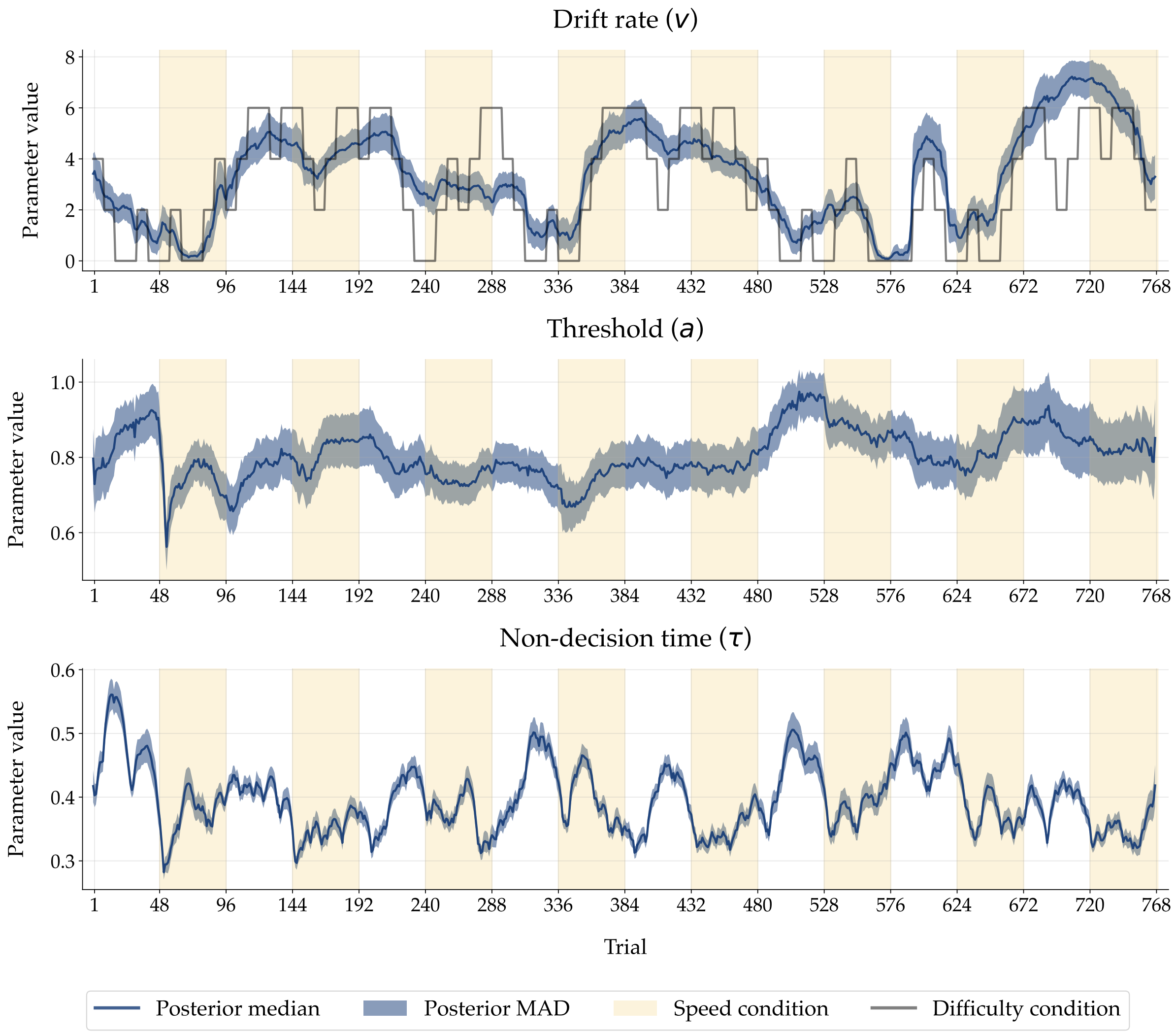

Fig. G39 Posterior parameter trajectory inferred with the best fitting NSDDM of participant 9 (a Lévy flight DDM in this case) for all three DDM parameters (drift rate, threshold, and non-decision time) separately. The yellow shaded areas indicate trials where speed was emphasised over accuracy and blank white area indicated where the opposite was asked for. In the top panel, the task difficulty levels sequence is depicted in black lines.

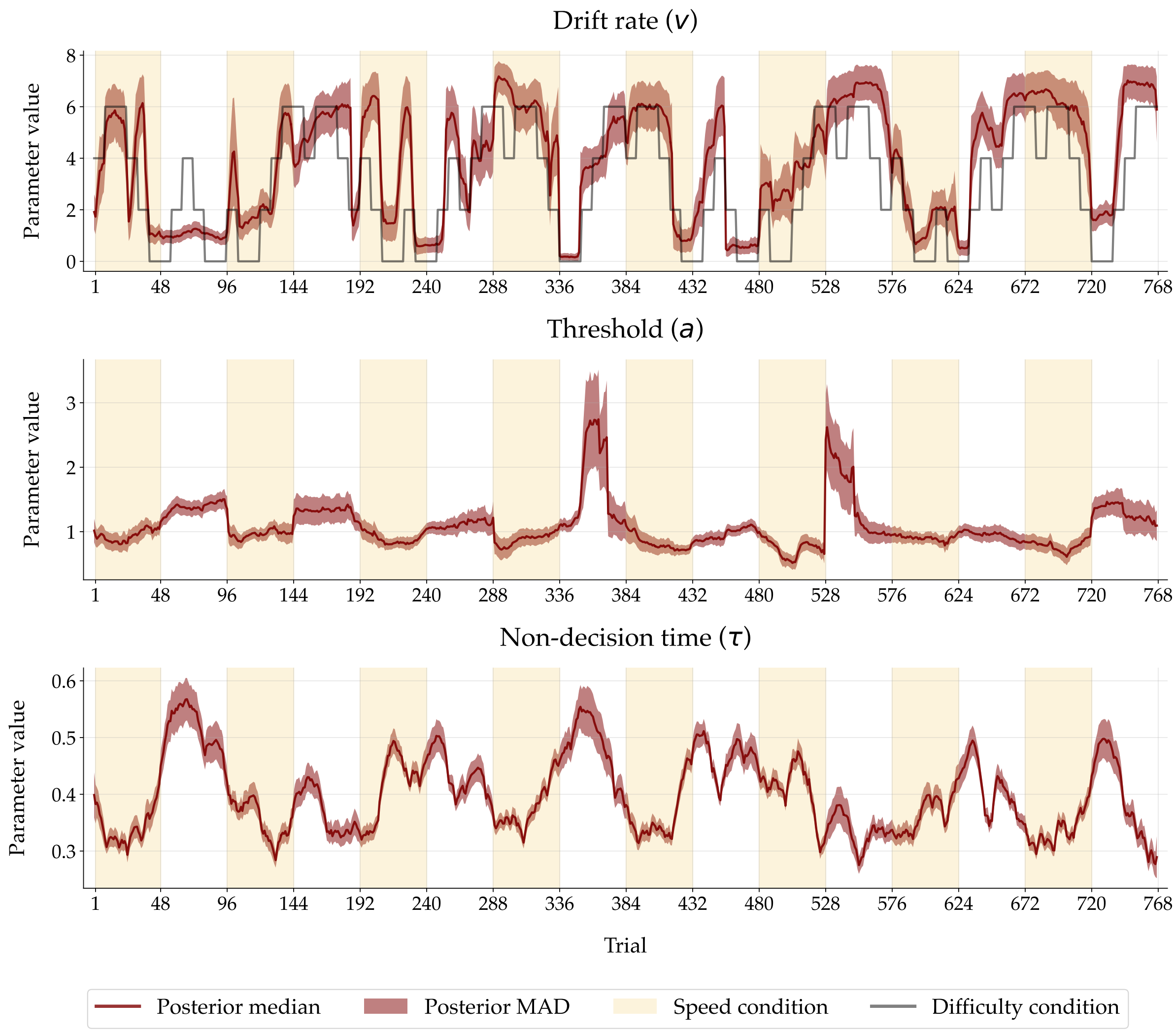

Fig. G40 Posterior parameter trajectory inferred with the best fitting NSDDM of participant 10 (a mixture random walk DDM in this case) for all three DDM parameters (drift rate, threshold, and non-decision time) separately. The yellow shaded areas indicate trials where speed was emphasised over accuracy and blank white area indicated where the opposite was asked for. In the top panel, the task difficulty levels sequence is depicted in black lines.

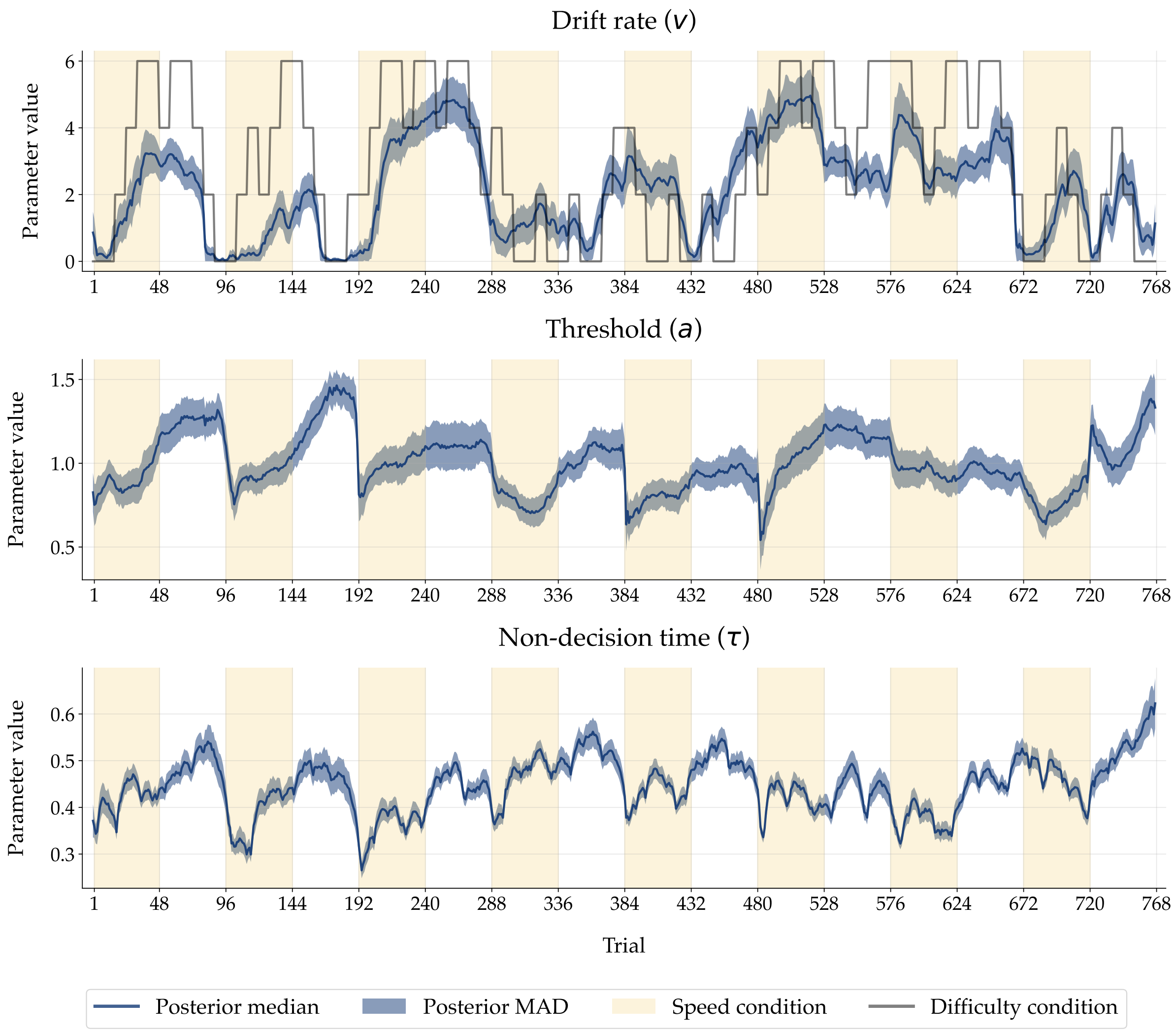

Fig. G41 Posterior parameter trajectory inferred with the best fitting NSDDM of participant 12 (a Lévy flight DDM in this case) for all three DDM parameters (drift rate, threshold, and non-decision time) separately. The yellow shaded areas indicate trials where speed was emphasised over accuracy and blank white area indicated where the opposite was asked for. In the top panel, the task difficulty levels sequence is depicted in black lines.

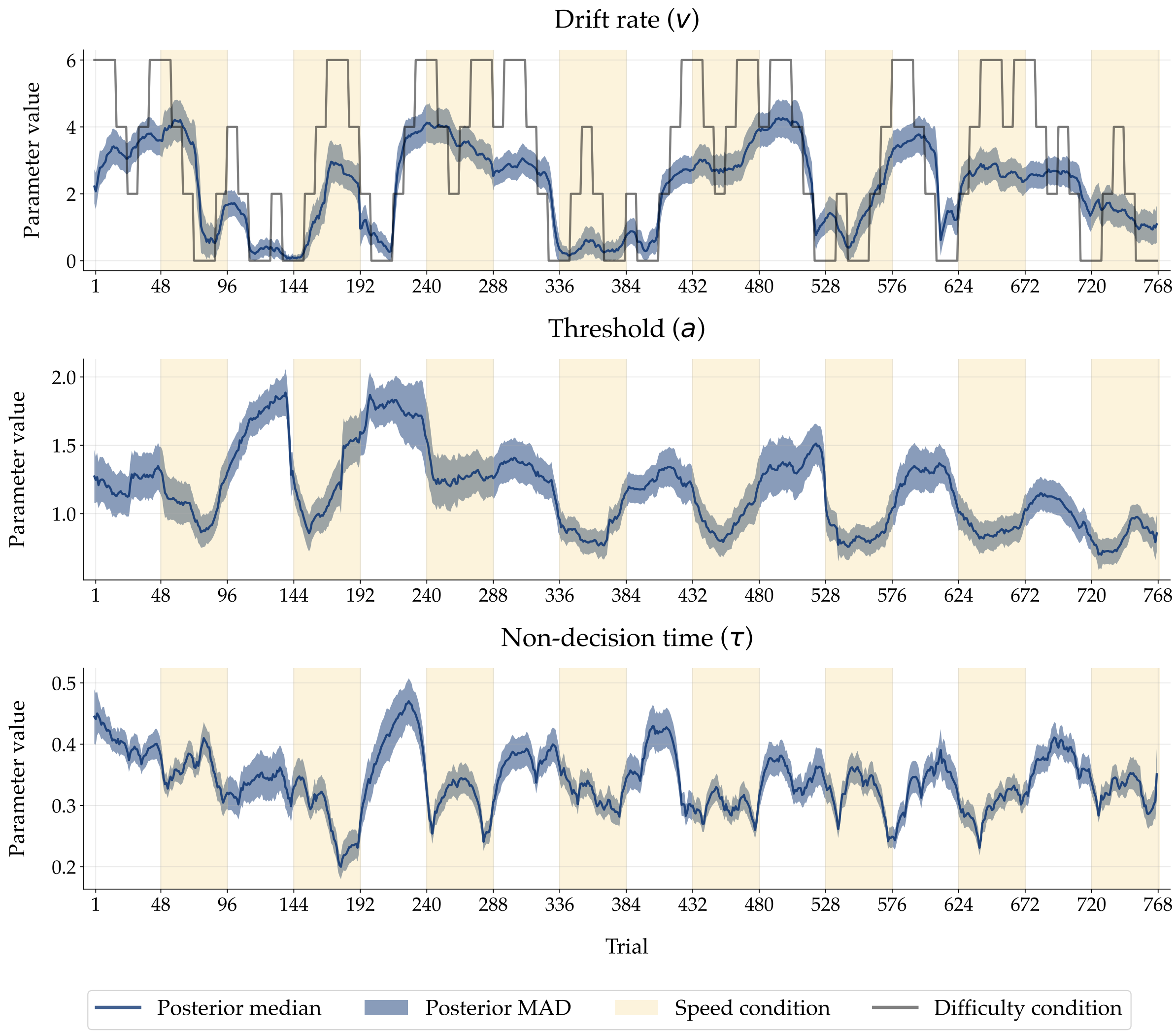

Fig. G42 Posterior parameter trajectory inferred with the best fitting NSDDM of participant 13 (a Lévy flight DDM in this case) for all three DDM parameters (drift rate, threshold, and non-decision time) separately. The yellow shaded areas indicate trials where speed was emphasised over accuracy and blank white area indicated where the opposite was asked for. In the top panel, the task difficulty levels sequence is depicted in black lines.

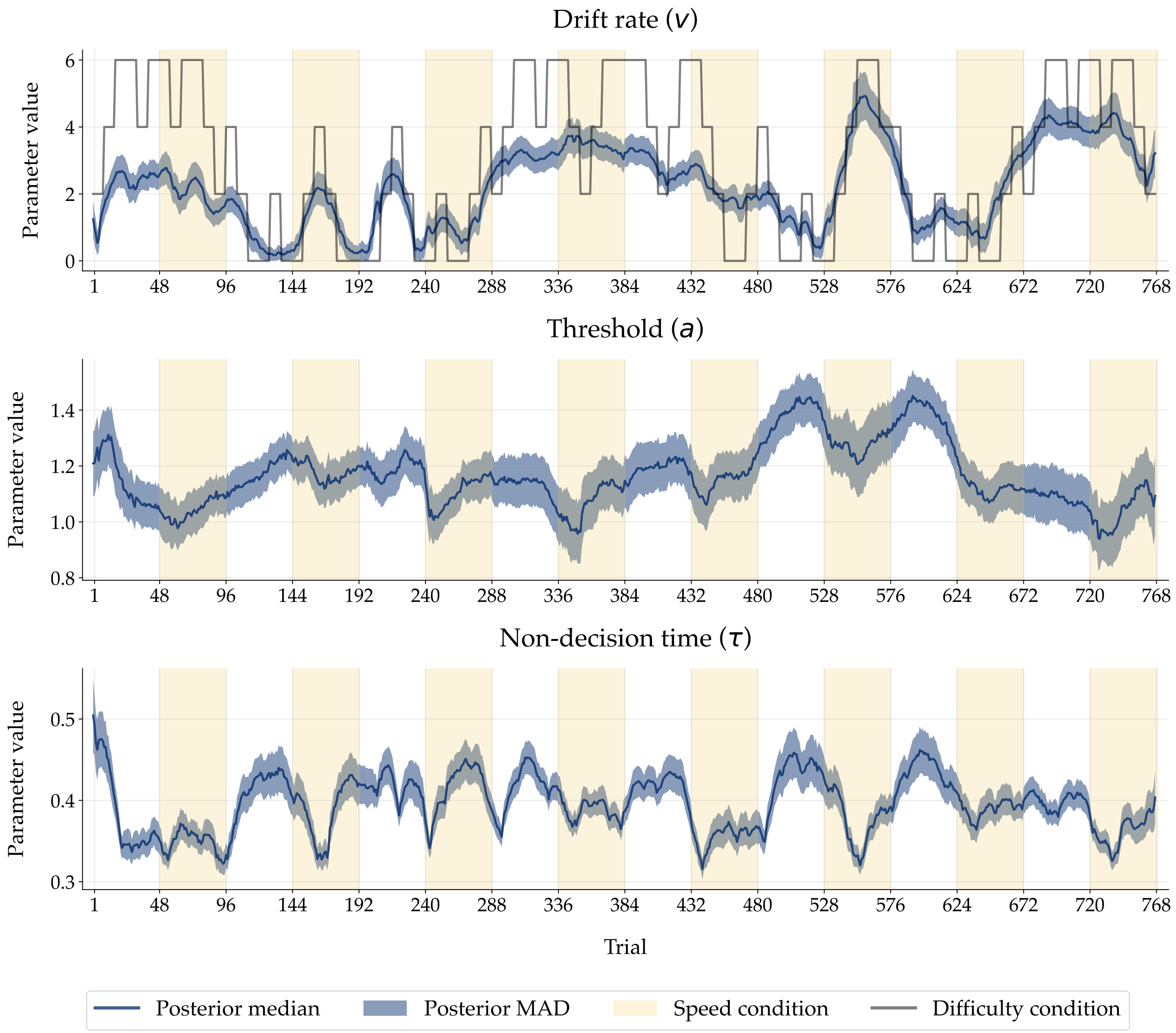

Fig. G43 Posterior parameter trajectory inferred with the best fitting NSDDM of participant 14 (a Lévy flight DDM in this case) for all three DDM parameters (drift rate, threshold, and non-decision time) separately. The yellow shaded areas indicate trials where speed was emphasised over accuracy and blank white area indicated where the opposite was asked for. In the top panel, the task difficulty levels sequence is depicted in black lines.
